# The herbal pair of *Smilax glabra* Roxb. and *Ficus hirta* Vahl. improves exercise performance by regulating mitochondrial function via the adiponection receptors-mediated AMPK signaling pathway

**DOI:** 10.3389/fnut.2026.1782001

**Published:** 2026-06-15

**Authors:** Ya-Nan Bao, Wen-Hao Wang, Hai-Xia Yang, Lei Qian, Xue Sui, Meng Wang, Bo-Wei Su, Fang-Yuan Chen, Xiao-Hui Cui, Jia-Yi Lao, Rui-Kang Wen, Hong-Yu Ji, Wen-Juan Hu, Lin Wen, Hui-Wen Ren, Zhi-Lin Luan

**Affiliations:** 1 Advanced Institute for Medical Sciences, Dalian Medical University, Dalian, China; 2 Key Laboratory of Sports Human Science in Liaoning Province, College of Physical Education, Liaoning Normal University, Dalian, China; 3 Dalian Key Laboratory for Nuclear Receptors in Major Metabolic Diseases, Dalian, China

**Keywords:** adiponectin receptor, AMP-activated protein kinase (AMPK), exercise performance, mitochondria, peroxisome proliferator-activated receptor gamma coactivator 1-alpha (PGC-1α)

## Abstract

**Introduction:**

Exercise fatigue can significantly impair physical function, while herbal medicines exhibit broad application prospects in anti-fatigue research. The herbal pair of *Smilax glabra* Roxb. and *Ficus hirta* Vahl. (FSP) is well-known for its hepatoprotective, dampness-eliminating, and detoxifying effects. However, its mechanism of action in improving exercise performance remains unclear.

**Methods:**

The impact of FSP on exercise performance was assessed through motor behavioral tests and monitoring of energy metabolism in both resting and exercise states. The anti-fatigue effects were evaluated by measuring fatigue-related biochemical markers in serum and urine using biochemical assay kits. Transcriptome sequencing of liver tissues from the animal model was conducted, and differentially expressed genes as well as significantly enriched pathways were identified via GO and KEGG enrichment analyses. Subsequently, a multi-parameter evaluation system was established, and in conjunction with molecular docking technology, two potential key active components in FSP were screened. Finally, their mechanisms of action were further verified using animal and cell-based experiments.

**Results:**

This study confirmed that FSP intervention significantly improved exercise performance in mice, accelerated lactate clearance, enhanced energy metabolism, and increased hepatic and muscular glycogen reserves, while alleviating oxidative stress and reducing systemic inflammation levels. Mechanistic investigations demonstrated that FSP activated the expression of adiponectin receptors (AdipoR1 and AdipoR2) in mouse liver, promoting the overexpression of downstream phosphorylated AMPK (p-AMPK) and PGC-1α, thereby regulating mitochondrial function and lipid metabolism. Furthermore, the study identified taxifolin and apigenin as the potential primary active components in FSP responsible for modulating the AdipoRs-AMPK pathway, with evidence of a synergistic effect between these two compounds.

**Conclusions:**

FSP regulates mitochondrial function through the AdipoRs-AMPK signaling pathway to exert anti-fatigue effects and enhance exercise performance.

## 1 Introduction

Fatigue, as a complex pathophysiological state, is categorized into two primary types: central and peripheral (1). Peripheral fatigue (physical fatigue) is more frequently encountered in daily life and is typically induced by prolonged or high-intensity physical activity. Its underlying mechanisms primarily involve the sustained depletion of energy substrates, accumulation of metabolites such as lactate, blood ammonia, and blood urea nitrogen (2), release of inflammatory factors (3), and excessive production of mitochondrial reactive oxygen species (ROS) (4). These factors collectively lead to impaired muscle contraction capacity and diminished exercise performance. Therefore, on the basis of adhering to scientific exercise (such as regular aerobic exercise) to enhance athletic performance, the appropriate use of nutritional or pharmaceutical supplements is also regarded as an important strategy for combating fatigue (5).

In recent years, various herbal medicines and their extracts have demonstrated significant potential in alleviating exercise-induced fatigue (6). *Smilax glabra* Roxb. and *Ficus hirta* Vahl. (FSP) has been recognized as a classical herbal pair originating from the Lingnan region of China, with efficacy derived from two core medicinal plants (plant information can be verified via https://www.worldfloraonline.org/). The first component, *Smilax glabra* Roxb. (SGB), known as Tufuling in Chinese, was first documented in the Ben Cao Jing Ji Zhu during the Southern and Northern Dynasties (420–589 AD) (7). Its yellowish-brown, irregular rhizome part is the medicinal portion. Modern pharmacological studies have revealed that SGB contains over 200 chemical components and exhibits a broad spectrum of pharmacological activities, including anti-infective, antitumor, anti-inflammatory, antioxidant, and cardiovascular protective effects (7). The other component, *Ficus hirta* Vahl. (FV), was first recorded for its medicinal value in the Qing Dynasty in Shengcao Yaoxing Beiyao (8). Known as Wuzhimaotao in Chinese due to its five-lobed leaf shape and small peach-like mature fruits, its rhizome possesses a distinctive aroma and serves as an important medicinal food. Currently, FV is primarily utilized for its protective activities such as antioxidant, anti-inflammatory, and antifungal effects (8). Notably, its aqueous extract has been shown to exhibit significant hepatoprotective effects *in vivo* (9). While the herbal pair is traditionally used for hepatoprotection, anti-inflammatory, and antioxidant purposes, its role in improving exercise performance and the underlying molecular mechanisms remain unreported.

Adiponectin (APN), an adipocytokine specifically secreted by adipose tissue, contains a highly conserved C-terminal globular domain homologous to complement factor C1q (gAd) (10). This structural domain enables its binding to and activation of the membrane receptors AdipoR1 and AdipoR2 (11). AdipoR1 is widely expressed in the liver and skeletal muscle, while AdipoR2 is predominantly expressed in the liver (12). Upon binding to these receptors, adiponectin initiates the AMP-activated protein kinase (AMPK) (13) signaling pathway, thereby regulating fatty acid oxidation and glucose metabolism. Notably, beyond its role as an energy sensor coordinating glucose and lipid homeostasis, AMPK directly promotes mitochondrial biogenesis and maintains mitochondrial homeostasis (14), which is crucial for mitigating exercise-induced energy stress (15). Multiple experimental studies have demonstrated that elevated plasma adiponectin levels significantly improve hepatic metabolic function and enhance skeletal muscle metabolic activity (16, 17), with a notable bidirectional regulatory relationship existing between adiponectin secretion and mitochondrial functional status (18). This provides a key target for developing exercise supplements. Based on preliminary animal experiments where we observed that FSP significantly enhanced exercise capacity in mice, combined with initial RNA-Seq data analysis, we hypothesize that FSP may exert its anti-fatigue effects through the AdipoRs (AdipoR1 and AdipoR2)-mediated AMPK signaling pathway. This hypothesis will be systematically validated in subsequent experiments.

The chemical composition of herbal medicine systems is highly complex and diverse, making the identification of their pharmacodynamic material basis a current research priority (19). Through analysis of literature and database resources related to the two core herbs in FSP, taxifolin and apigenin have attracted attention due to their notable bioactivities. Further bioinformatics (molecular docking) studies indicate that both compounds exhibit potential for effectively binding to AdipoR1 and AdipoR2. Considering the chemical composition profile of FSP, we preliminarily propose that taxifolin and apigenin, as important monomeric constituents present in FSP, may play a critical role in its overall efficacy. Verification of this hypothesis has been incorporated as a key component into the subsequent experimental design. In summary, this study aims to systematically validate the molecular mechanism by which FSP exerts its anti-fatigue effects via the AdipoRs-AMPK axis and to identify its key active constituents, thereby providing a scientific basis for the application of traditional herbal compatibility.

## 2 Materials and methods

### 2.1 Materials preparation

#### 2.1.1 Plant material and extraction preparation

The dried medicinal herbs used in the experiment (20 g of *Smilax glabra* Roxb. and 20 g of *Ficus hirta* Vahl.) were obtained from Tongrentang Pharmacy (Liaoning, China). The herbs were decocted twice in a 1:1 mass ratio, with double-distilled water added at a volume 10 times the weight of the herbs (10 ml of solvent per gram of herbal material) for each decoction. Prior to the first decoction, the herbs were soaked for 90 min. After boiling, the heat was reduced to a low flame, and the herbs were continuously decocted for 2 h. Subsequently, the decoction was cooled and filtered to collect the liquid. The procedure for the second decoction was identical to that of the first. The liquid from both decoctions was combined and concentrated to 167 ml via rotary evaporation, followed by lyophilization using a freeze dryer (B04-20FB0005, Xinzhi Biotechnology Co., Ltd., Ningbo, China) to produce a dry powder (5.7829 g). The dry powder was sealed and stored at 4 °C. Prior to use, it was dissolved in double-distilled water and filtered through a 0.22 μm membrane.

#### 2.1.2 Monomer preparation

Taxifolin (CAS No. 480-18-2) with a purity of 99.1% was obtained from APExBIO (Houston, TX, USA). Apigenin (CAS No. 520-36-5) with a purity ≥98.0% was purchased from MACKLIN (Shanghai, China).

### 2.2 Animals and experimental design

The experimental protocol was approved by the Animal Ethics Committee of Dalian Medical University (Approval No. XL250815303), and all procedures were conducted in accordance with established guidelines for animal research. Adult male C57BL/6 mice aged 7–8 weeks (21–23 g) used in the experiments were obtained from Changsheng Biotechnology Co., Ltd. (Liaoning, China). Following a three-day acclimation period under standard environmental conditions (12 h light/dark cycle, 40–70% humidity, temperature 23 ± 1 °C), all mice proceeded to subsequent experimental procedures. (All mice that underwent post-gavage experimental procedures were in normal condition, with no instances of mechanical injury resulting from the gavage procedure).

#### 2.2.1 FSP animal model

**Figure 1:**
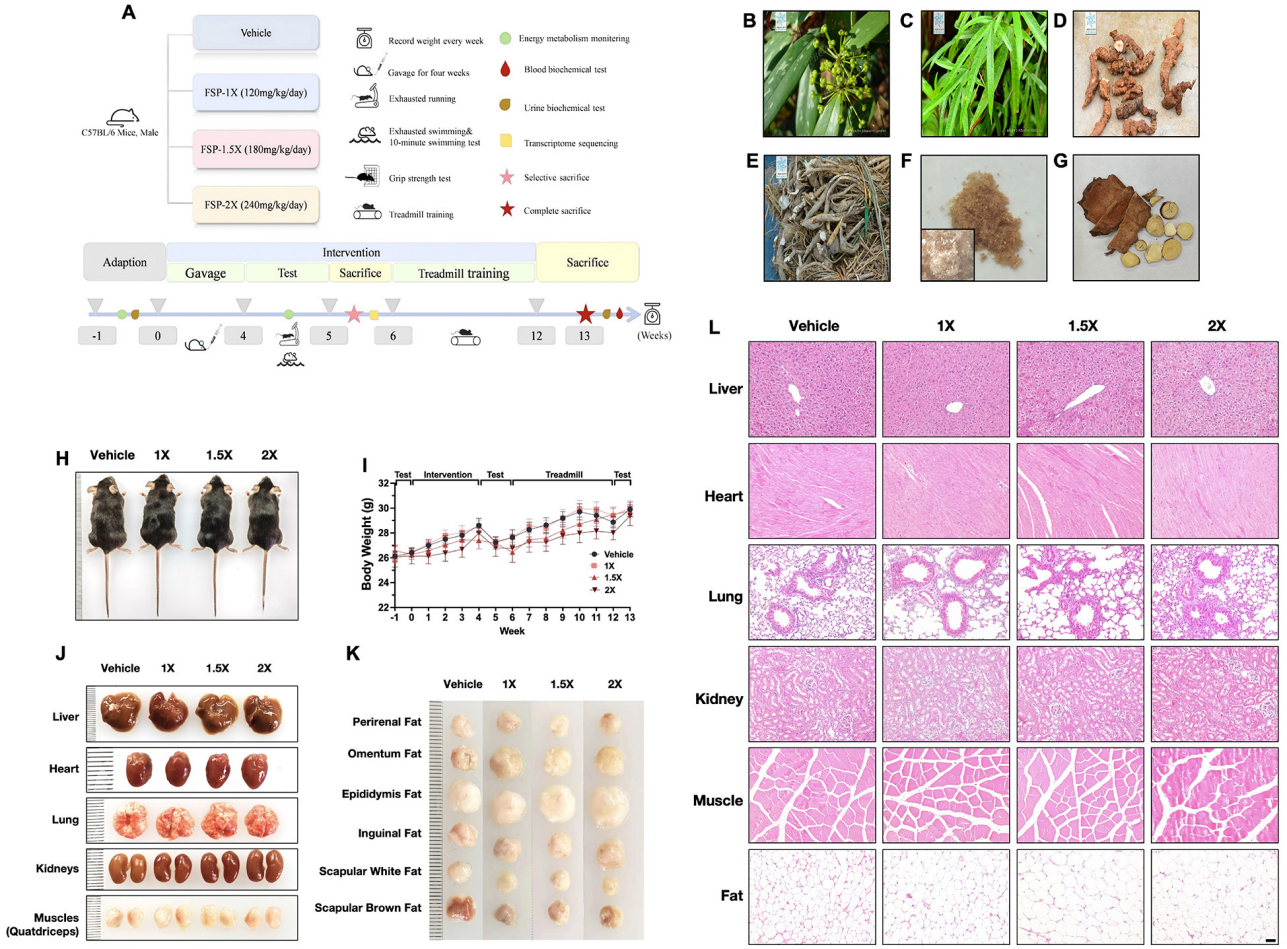
Composition, experimental design, and basic observations of FSP. **(A)** FSP-animal experimental design; **(B–G)** FSP components: **(B)** *Smilax glabra* Roxb. (plant) Source: https://ppbc.iplant.cn/tu/605969; **(C)** *Ficus hirta* Vahl. (plant) Source: https://ppbc.iplant.cn/tu/19711513; **(D)** Rhizome of *Smilax glabra* Roxb. Source: https://ppbc.iplant.cn/tu/5400091; **(E)** Rhizome of *Ficus hirta* Vahl. Source: https://ppbc.iplant.cn/tu/12781180; **(F)** Images of dried rhizomes of *Smilax glabra* Roxb. and *Ficus hirta* Vahl.; **(G)** Lyophilized powder of *Smilax glabra* Roxb. and *Ficus hirta* Vahl.; **(H)** gross morphology of mice after FSP administration; **(I)** Weekly body weight monitoring in FSP groups; **(J, K)** Organ and adipose tissue observations in FSP groups; **(L)** H & E staining of major organs and adipose tissues in FSP groups (Scale bar: 20 μm, Total magnification: 200x). Source: Images **(B)–(E)** from the Plant Photo Bank of China (https://ppbc.iplant.cn/), reproduced under CC BY-NC-ND 4.0 license.

Assuming an average mouse body weight of 20 g and applying equivalent dose conversion based on a standard human body weight of 50 kg, the daily administration dose for mice was determined to be 2.31 mg. The mice were randomly divided into four groups using a random number table method (*n* = 8 per group): vehicle group, FSP-1X group (120 mg/kg/d), FSP-1.5X group (180 mg/kg/d), and FSP-2X group (240 mg/kg/d). During group allocation, it was ensured that there were no statistically significant differences in the initial mean body weight among the groups, in order to eliminate the potential influence of individual variability on the experimental outcomes. The vehicle group received an equivalent volume of drug solvent (double-distilled water) proportional to individual body weight. Following a 3-day acclimation period, both control and FSP-treated mice underwent continuous intragastric administration for 4 weeks, with weekly body weight monitoring. The detailed experimental workflow is presented in Figure 1A.

#### 2.2.2 Monomeric animal model

**Figure 2:**
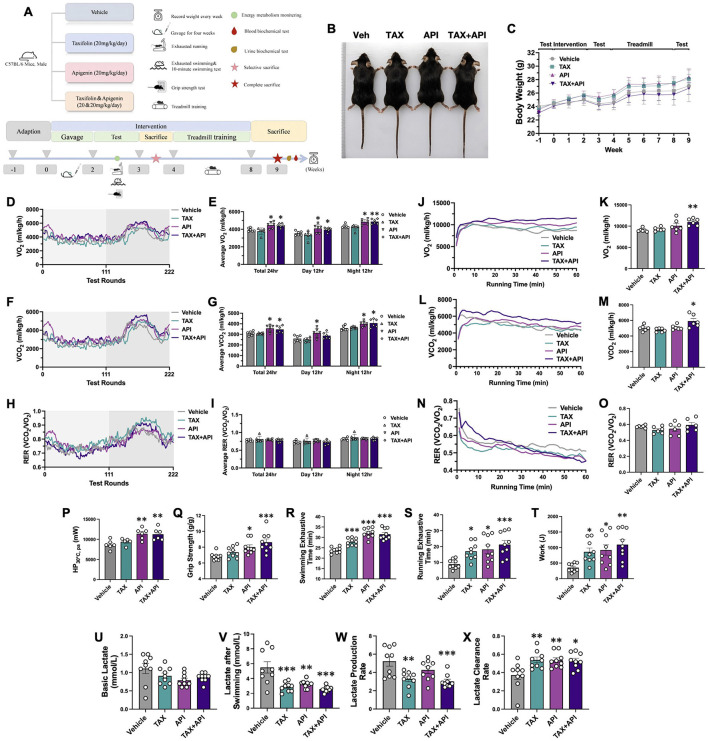
Synergistic enhancement of exercise performance by combined taxifolin and apigenin intervention in mice **(A)** Schematic diagram of animal experimental procedure for monomeric compounds; **(B)** Apparent comparative image of mice after monomeric gavage; **(C)** Dynamic weight change records of mice in each group during the experiment; **(D–I)** VO2, VCO2, and RER of four mouse groups at rest (*n* = 6); **(J–O)** VO2, VCO2, and RER of four mouse groups during exercise (*n* = 6); **(P)** BMR of mice in each group at 30 °C constant temperature (*n* = 6); **(Q–T)** Exercise behavioral indicators (forelimb grip strength, exhaustive swimming, exhaustive running, mechanical work) of mice after monomeric gavage (*n* = 9). **(U–X)** 10-min swimming test (Lactate test). Results are expressed as mean ± SEM. *p < 0.05, **p < 0.01, ***p < 0.001 compared to the vehicle group.

The mice were again randomly divided into 4 groups (*n* = 8 per group) using a random number table, in order to eliminate the influence of individual differences on the experimental results: vehicle group, taxifolin group (20 mg/kg/d) (20), apigenin group (20 mg/kg/d) (21), and taxifolin + apigenin group (20 mg/kg/d & 20 mg/kg/d). Based on solubility data provided by MedchemExpress (https://www.medchemexpress.cn), and with the vehicle group receiving an equivalent volume of drug solvent proportional to individual body weight, all mice underwent 2 weeks of intragastric administration before subsequent experimental investigations. The specific experimental procedure is shown in Figure 2A.

#### 2.2.3 Blinding implementation procedure

In this study, a double-blind method was adopted for data collection and analysis. The specific procedure was as follows: an experimenter not involved in subsequent data detection was responsible for the preparation of the drug and solvent, and coded the mice according to the randomization results. Another experimenter, who was in charge of body weight monitoring, behavioral tests, and energy metabolism measurements, was aware only of the mouse codes and remained blinded to the treatment allocation of each group. After all data were collected, a third experimenter with no direct involvement in the animal experiments performed the statistical analysis without knowledge of the group assignments.

### 2.3 Exercise performance test

To ensure standardization of experimental conditions, all mouse experiments were conducted between 14:00 and 17:00.

#### 2.3.1 Forelimb grip strength test

Forelimb grip strength was assessed with a grip strength meter (ZS-ZL, Zhongquanchuang Technology Development Co., Ltd., Beijing, China). Mice were allowed to grasp a horizontally positioned metal grid with forepaws only before being drawn backward steadily by the tail base until grip release. Peak grip force was recorded during this procedure. Each mouse underwent five consecutive trials separated by 3-min rest intervals.

#### 2.3.2 Exhaustive running test

Exercise capacity was evaluated using an SA101B animal treadmill (Saien Biotechnology Co., Ltd., Jiangsu, China). Following a 3-day acclimation protocol (15 min/day at 10 m/min, 0° incline), mice fasted for 4 h underwent testing with a progressive acceleration regimen (initial speed 12 m/min, increasing by 2 m/min every 2 min until reaching 22 m/min, 12° incline). Exhaustion was defined as failure to resume running within 10 s of continuous grid contact, with total running duration recorded.

#### 2.3.3 Exhausted swimming training

Prior to the experiment, mice were fasted for 4 h. A constant load equivalent to 5% of their body weight was attached to the tail of each mouse. Each mouse was then individually placed in a rectangular water tank (80 cm long, 80 cm wide, 30 cm deep, water temperature 37 ± 0.5 °C). The mice were observed for signs of limb incoordination and inability to return to the water surface for breathing. The endpoint criterion was defined as the mouse's failure to resurface within 10 s after its head submerged.

#### 2.3.4 10-min swimming test (Lactate test)

Each mouse was individually placed in a rectangular water tank and subjected to a 10-min forced swimming session without additional load. Blood lactate levels were measured using a lactate analyzer (YST-1, Yangzhou Beifu Medical Technology Co., Ltd., Yangzhou, China) at three time points: before swimming, immediately after swimming, and 20 min after the swimming session.

#### 2.3.5 Aerobic exercise training

Mice underwent a 6-week aerobic training regimen with progressive intensity: 6 m/min for the initial 2 weeks, 8 m/min during the subsequent 2 weeks, and 10 m/min for the final 2 weeks. Each training session maintained a duration of 40 min.

### 2.4 Energy metabolism monitoring

#### 2.4.1 Resting metabolic measurement

The Oxymax-CLAMS system (Oxymax-CLAMS; Columbus Instruments, Columbus, OH, USA) was employed to assess resting metabolic rates in mice. Animals were individually maintained in metabolic cages under controlled conditions (24 °C, 35% humidity, 12h light/dark cycle) for measurement of oxygen consumption (VO2), carbon dioxide production (VCO2), heat generation, and respiratory exchange ratio (RER). Basal metabolic rate (BMR) was evaluated at 30 °C, with stable mid-experimental phase data utilized for subsequent analysis.

#### 2.4.2 Activity metabolic measurement

Each mouse was individually placed in a horizontal metabolic treadmill and underwent adaptive training prior to the experiment. At the start of the formal test, the treadmill speed was set at 10 m/min for a 60-min aerobic exercise session. The distal end of the metabolic treadmill was connected to the Oxymax Comprehensive Laboratory Animal Monitoring System to measure VO2, VCO2, and RER during exercise.

### 2.5 Biochemical assays

#### 2.5.1 Blood and urine biochemical index test

After all data collection was completed, the animals were euthanized by collecting tissues and serum under anesthesia. Biochemical indicators in serum and urine were measured using commercial kits (Nanjing Jiancheng Bioengineering Institute, Nanjing, China). Serum biochemical assays included alanine aminotransferase (ALT), aspartate aminotransferase (AST), alkaline phosphatase (ALP), creatine kinase (CK), total protein (TP), blood urea nitrogen (BUN), creatinine (Cr), uric acid (UA), total cholesterol (TC), triglycerides (TG), glucose (GLU), lactate dehydrogenase (LDH), and blood ammonia (E-BC-K145-S, Elabscience, Wuhan, China). Urinary biochemical analyses included Cr and UA. Absorbance at respective peak wavelengths was measured using a multifunctional microplate reader (Thermo Fisher Scientific, Waltham, MA, USA). Sodium and potassium ion concentrations in urine were determined using an automated electrolyte analyzer (HC-9885, Haichuang Medical Equipment Co., Ltd., Shenzhen, China). Urine osmolality was measured with a freezing-point osmometer (Advanced Instruments Technology Co., Ltd., Norwood, MA, USA).

#### 2.5.2 Tissue glycogen measurement

The glycogen assay kit was purchased from Nanjing Jiancheng Bioengineering Institute (Nanjing, China). Liver and muscle tissue samples from mice were processed in strict accordance with the kit instructions. Processed samples were analyzed using a multifunctional microplate reader (Thermo, Waltham, MA, USA) to obtain quantitative glycogen content measurements.

### 2.6 Histological analysis

#### 2.6.1 Hematoxylin and eosin staining (H & E)

Mouse tissue samples (heart, liver, lung, kidney, muscle, and perirenal fat) were fixed in 4% paraformaldehyde (PFA) at 4 °C for 24 h. Following dehydration and paraffin embedding, 4-μm sections were prepared. Staining was performed according to the H & E kit protocol (Solarbio, Beijing, China), with subsequent observation under an Olympus BX41 (Tokyo, Japan) optical microscope.

#### 2.6.2 Immunohistochemistry (IHC)

After dewaxing the 4 μm paraffin sections, antigen retrieval was performed using citrate buffer (3 min at high heat, 10 min at low heat). After natural cooling, sections were washed three times with PBS for 5 min each. Endogenous peroxidase activity was blocked by incubation with 3% H2O2 in a sealed humidified chamber at room temperature for 10 min, followed by three PBS washes and 1-h blocking at room temperature. Subsequently, sections were incubated overnight at 4 °C with primary antibodies against PGC1α, COX IV, and TFAM at a dilution of 1:200. The following day, primary antibodies were recovered, and sections were washed three times with PBS before incubation with secondary antibodies (Zhongshan Jinqiao, Beijing, China) for 1–2 h. After final washing with PBS (three times), color development was performed using DAB (Zhongshan Jinqiao, Beijing, China), followed by counterstaining of nuclei with hematoxylin. After dehydration, sections were mounted with neutral balsam (Solarbio, Beijing, China), dried at 37 °C, and observed under an optical microscope for image acquisition. Quantitative analysis of positively stained areas was performed using ImageJ software (v1.45; NIH, Bethesda, MD, USA; https://imagej.net/ij/).

### 2.7 Transcriptome sequencing

Sample collection was conducted immediately after the completion of FSP gavage treatment, and the sequencing of mouse liver tissues along with subsequent bioinformatics analysis was entrusted to Novogene Bioinformatics Technology Co., Ltd. (Beijing, China). Total RNA was extracted from mouse liver tissue using Trizol reagent (Vazyme, Nanjing, China) in accordance with the manufacturer's protocol, followed by DNase I (Takara, Kusatsu, Japan) treatment to eliminate genomic DNA contamination. After rigorous quality control assessing RNA integrity and purity, high-quality RNA samples meeting the standards (OD260/280 ratio between 1.8 and 2.2) were selected for subsequent experiments. Sequencing library preparation and high-throughput sequencing were performed on the Illumina NovaSeq 6000 platform. Data analysis was conducted on the Novogene MagicPlus platform (https://magic-plus.novogene.com/), and the RNA-Seq data were deposited in the GEO database. Finally, Kyoto Encyclopedia of Genes and Genomes (KEGG) enrichment analysis was employed to explore the biochemical metabolic and signal transduction pathways associated with the target genes.

### 2.8 Cell experiment

#### 2.8.1 Cell culture

HepG2 cells were cultured in DMEM high-glucose medium (Procell Life Science, Wuhan, China) supplemented with 10% fetal bovine serum (Gibco, Thermo, Waltham, MA, USA), 100 IU/ml penicillin (Beyotime, Shanghai, China), and 100 μg/ml streptomycin (Beyotime, Shanghai, China). The cells were maintained at 37 °C in a 5% CO_2_ incubator (Thermo, Waltham, MA, USA).

#### 2.8.2 Cell treatments

First, experimental mice were subjected to a 4-week gavage treatment (ddH2O2/FSP at a dose of 120 mg/kg/d). Blood was subsequently collected via orbital puncture and processed by centrifugation and filtration to obtain herbal medicine-containing serum for *in vitro* cell culture experiments. Four experimental groups were established: vehicle group (veh-10% serum), FSP low-concentration serum group (FSP-10% serum), FSP medium-concentration serum group (FSP-15% serum), and FSP high-concentration serum group (FSP-20% serum).

Four additional monomer intervention groups were established: vehicle group (DMSO), taxifolin group (20 μm) (20), apigenin group (20 μm) (22), and taxifolin + apigenin group (20 μm & 20 μm). All cell cultures were maintained under standard conditions (37 °C, 5% CO_2_) for 24 h before collective harvesting for subsequent analysis.

### 2.9 Molecular biology experiment

#### 2.9.1 Western blotting

Western blot analysis was performed to compare protein expression profiles in liver tissues from different experimental groups and in HepG2 cells. Antibodies used in this study were obtained from ABclonal Technology Co., Ltd. (Wuhan, China), Proteintech Group, Inc (Wuhan, China) and Cell Signaling Technology, Inc. (Danvers, MA, USA). The specific antibodies used were as follows: AdipoR1 (Abclonal, A1509), AdipoR2 (Abclonal, A12777), p-AMPK (Abclonal, AP1441), AMPK (Abclonal, A1229), p-LKB1 (Abclonal, AP0601), LKB1 (Abclonal, A22636), PGC1α (Proteintech, 66369-1-Ig), SIRT1 (Proteintech, 13161-1-AP), LDHB (Proteintech, 14824-1-AP), COXIV (Cell Signaling Technology, 3E11), TFAM (Abclonal, A3173), NRF2 (Proteintech, 16396-1-AP), HO-1 (Abclonal, A19062), TNFα (Proteintech, 80258-6-RR), COX2 (Abclonal, A3560), and β-actin (Proteintech, 20536-1-AP). Total protein was extracted from the samples using RIPA lysis buffer. After centrifugation at 12,000 rpm at 4 °C, the supernatant was collected. Protein quantification was performed using a BCA protein assay kit (Thermo, Waltham, MA, USA). Depending on the molecular weight of the target protein, 10% or 12% SDS-PAGE gel electrophoresis was used for separation, followed by transfer onto a PVDF membrane (Merck Millipore, Shanghai, China). After blocking with 5% non-fat milk, the membrane was incubated overnight at 4 °C with the corresponding primary antibody. Following washing with TBST, the membrane was incubated with a horseradish peroxidase-conjugated secondary antibody (1:5,000) at room temperature for 1–2 h. Finally, the membrane was developed using an ECL chemiluminescent substrate (Epizyme, Shanghai, China), and images were captured using a Tanon 5200 chemiluminescent imaging system (Shanghai, China).

#### 2.9.2 Reverse transcription-quantitative PCR (RT- qPCR)

Total RNA was isolated from mouse liver tissues and HepG2 cells using Trizol reagent (Vazyme, Nanjing, China). The concentration and purity of the extracted RNA were determined using a semi-automated protein/nucleic acid analyzer (Thermo Fisher Scientific, Waltham, MA, USA). Following reverse transcription of RNA into cDNA, quantitative real-time PCR was performed on a real-time PCR system (Thermo Fisher Scientific, Waltham, MA, USA), with β-actin serving as the internal reference gene. Primer sequences are detailed in Supplementary Table S1.

### 2.10 Cell viability assay

Cells were seeded in 96-well plates at a density of approximately 5,000 cells per well. After cell adhesion, the assay wells were treated with drug-containing serum at varying concentrations, while blank control wells (containing drug and CCK-8 solution but no cells) and negative control wells (containing cells, drug solvent, and CCK-8 solution but no drug compounds) were established simultaneously. Following drug administration, the plates were incubated for 24 h. Subsequently, CCK-8 solution (Meilun, < city>Dalian < /city>, China) was added at a 1:10 ratio, followed by additional incubation for 1–2 h. Absorbance at 450 nm was measured using a multifunctional microplate reader to assess cell proliferation activity.

### 2.11 Inhibitor experiments

Compound C (10 μm, B3252, APExBIO, Shanghai, China) was added to both the vehicle group and the FSP high-concentration serum group (FSP-20% serum). After 24 h of incubation, the samples were collected for subsequent use.

### 2.12 Cellular immunofluorescence staining

Cells were seeded onto coverslips and cultured with different concentrations of FSP serum or monomeric compounds. After incubation, cells were washed three times with PBS and fixed with 4% PFA (Servicebio, Wuhan, China) at room temperature for 20 min, followed by permeabilization with 0.5% Triton-X 100 (Sigma-Aldrich, St. Louis, MO, USA) for 10 min. Subsequently, cells were blocked with 5% BSA (prepared in PBS) at room temperature for 1 h. Following blocking, cells were incubated with primary antibodies at 4 °C overnight. Primary antibodies used (dilution ratio: 1:200) included: PGC1α, SIRT1, TFAM, and COXIV. Cells were then washed three times with PBS and incubated with Alexa Fluor 488/594-conjugated secondary antibodies (1:500, Zhongshan Jinqiao, Beijing, China). Nuclei were stained with 4′,6-diamidino-2-phenylindole (DAPI), and after mounting, images were captured using a fluorescence microscope (DM4B, Leica, Germany).

### 2.13 Mitochondrial function assessment

#### 2.13.1 Detection of mitochondrial membrane potential (MMP)

Mitochondrial membrane potential (ΔΨm) was assessed using a JC-1 staining kit (Beyotime, Shanghai, China). After 24 h of drug treatment, cells were stained according to the manufacturer's instructions and analyzed within 30 min. Images were acquired using a fluorescence microscope.

#### 2.13.2 Mitochondrial copy number

Genomic DNA was extracted from tissues and cells using a genomic DNA kit (Tiangen, Beijing, China). Following DNA concentration measurement, mitochondrial DNA copy number was detected by quantitative real-time PCR. Cytochrome b (mtCytb) and NADH dehydrogenase subunit 1 (mtND1) were used as markers for mitochondrial DNA replication, with β-actin serving as the internal reference gene for normalization. Experimental results were calculated and expressed using the 2^−^ΔΔC_T method. The primer sequences used were as follows:

##### 2.13.2.1 Human

mtCytb F5′-TCA CCA GAC GCC TCA ACC GC3′, R:5′-GCC TCG CCC GAT GTG TAG GA3′; mtND1 F:5′-GGA GTA ATC CAG GTC GGT-3′, R:5′-TGG GGT ACA ATG AGA GAG TAG G-3′

##### 2.13.2.2 Mouse

mtCytb F5′-ACC CGC CCC ATC CAA CAT CTC AT3′, R5′-TAC AAC CAT TTG CAG ACG CC3′; mtND1 F5′-TTG AGG CTC CGT TTG CGT GT3′, R5′-TGT GAG TGA TAG GGT AGG TGC-3′.

### 2.14 Measurement of intracellular reactive oxygen species (ROS)

Intracellular ROS levels in liver tissues and HepG2 cells were measured using DCFH-DA (Beyotime, Shanghai, China). All procedures were performed in strict accordance with the manufacturer's instructions. Fluorescence intensity was measured with a microplate reader (excitation wavelength: 488 nm, emission wavelength: 525 nm), and results were normalized to total protein content.

### 2.15 ATP detection

ATP levels in liver tissues and HepG2 cells were determined using an ATP assay kit (Beyotime, Shanghai, China). All experimental procedures were performed on ice and strictly followed the manufacturer's instructions. Luminescence was measured using a multifunctional microplate reader equipped with chemiluminescence detection capability, and the results were normalized to protein concentration.

### 2.16 Molecular docking

The crystal structures of adiponectin receptor 1 (PDB ID: 6KSO) and adiponectin receptor 2 (PDB ID: 5LWY) were downloaded from the Protein Data Bank (http://www.rcsb.org). The structural data of taxifolin (PubChem CID: 439533) and apigenin (PubChem CID: 5280443) were obtained from the PubChem database (https://pubchem.ncbi.nlm.nih.gov/). The structures of adiponectin receptors 1 and 2, taxifolin, and apigenin were prepared using AutoDockTools 1.5.7 (The Scripps Research Institute, La Jolla, CA, USA). The optimal docking conformations were visualized with PyMOL software (V 3.0.3, Schrödinger, LLc., NY, USA).

### 2.17 Cellular thermal shift assay (CETSA)

Aliquots of 100 μL HepG2 cell lysates were mixed and incubated separately with taxifolin and apigenin to achieve a final concentration of 50 μm for each compound. In the vehicle group, the cell lysate was incubated with DMSO, the solvent for both monomers. The mixtures were incubated at graded temperatures (37 °C, 41 °C, 44 °C, 49 °C, 53 °C, 57 °C, 61 °C, 65 °C, 69 °C, 73 °C) for 3 min. All samples were centrifuged at 12,000 rpm for 15 min to collect the supernatant for subsequent Western blot analysis.

### 2.18 Transmission electron microscopy(TEM)

Transmission electron microscopy observations were conducted by Jijia Biotechnology Co., Ltd. (Liaoning, China). After treatment, cell samples were fixed with 2.5% glutaraldehyde solution at room temperature. Cell pellets were gently collected and suspended in the fixative. Samples were first fixed for 2 h at room temperature in the dark, then stored at 4 °C prior to shipment for analysis. Mitochondrial distribution and ultrastructure were examined using a JEOL JEM-1400PLUS transmission electron microscope (Tokyo, Japan). Acquired images were subsequently analyzed.

### 2.19 Statistical analysis

All experimental data were statistically analyzed using GraphPad Prism 9 (GraphPad Software, Inc., San Diego, CA, USA). Measurement data are expressed as mean ± standard error of the mean (mean ± SEM). Comparisons between two groups were performed using unpaired Student's t-test, while one-way analysis of variance (ANOVA) was applied for multi-group comparisons. A threshold of *P* < 0.05 was considered statistically significant.

## 3 Results

### 3.1 FSP administration shows no adverse effects on basic physiological parameters in mice

The detailed experimental timeline is illustrated in Figure 1A. FSP consists of *Smilax glabra* Roxb. (Figures 1B, D) and *Ficus hirta* Vahl. (Figures 1C, E), which were processed into lyophilized powder (Figures 1F, G) for subsequent animal experiments. During the 4-week intragastric administration period, multiple basic physiological parameters were systematically evaluated. Results indicated that body shape and weight remained stable across all groups without significant fluctuations (Figures 1H, I). Although the liver-to-body weight ratio showed a slight increasing trend, no statistically significant differences were observed in the weights of major organs (Supplementary Table S2) or body fat percentage (Supplementary Figure S2) among the experimental groups. Metabolic cage monitoring further demonstrated no significant alterations in food intake, water consumption, urine volume, or urine osmolality before and after the intervention (Supplementary Figure S1). Moreover, gross anatomical observations (Figures 1J, K) and histological (H & E staining) analysis (Figure 1L) of the liver, other vital organs, and adipose tissues from all four groups revealed no notable pathological changes. Collectively, these findings demonstrate that FSP administration did not induce significant adverse effects on the overall health of the mice.

### 3.2 FSP supplementation alleviates fatigue, enhances energy metabolism and improves exercise performance in mice

**Figure 3:**
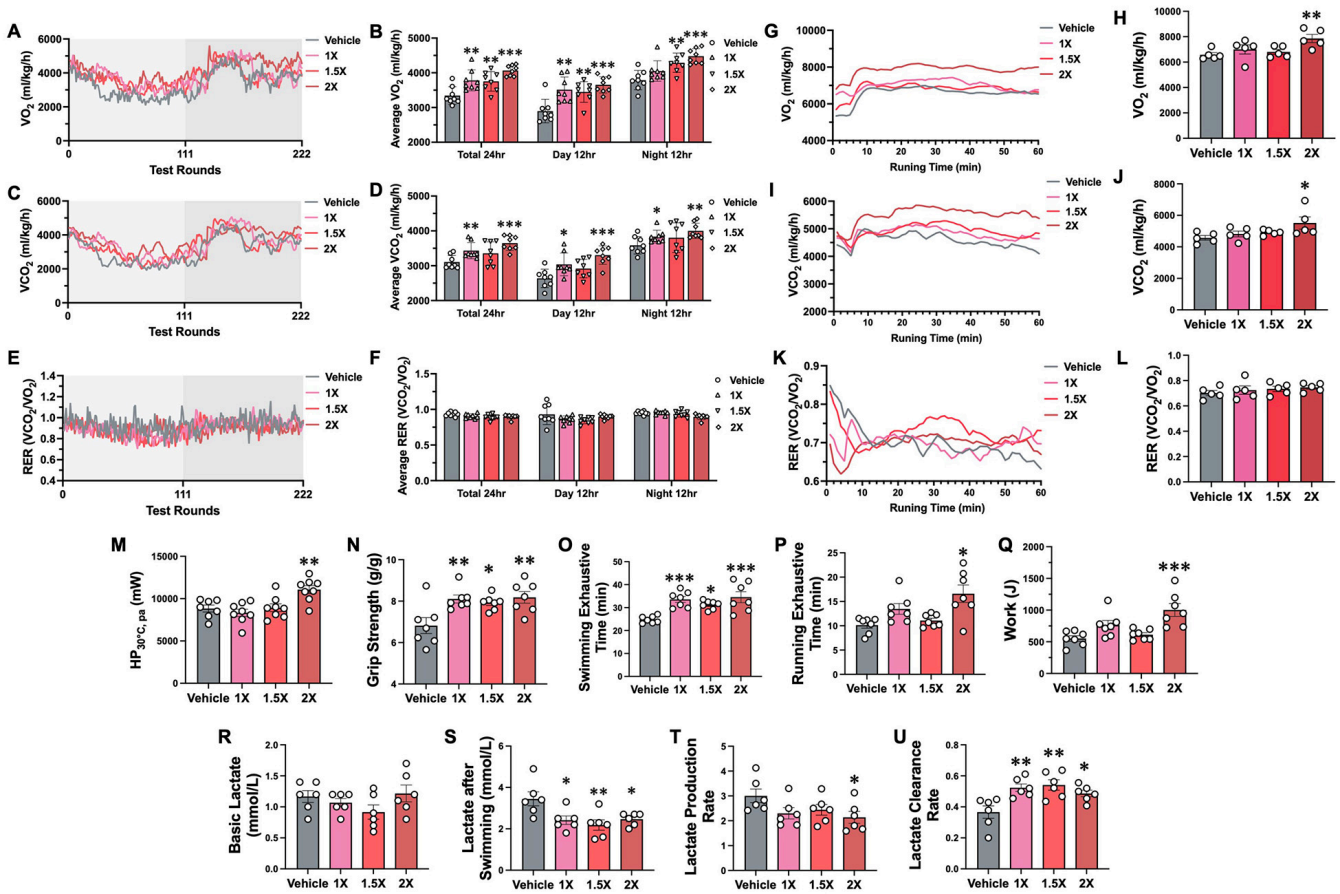
Effects of FSP on energy metabolism and motor behavioral performance in mice. **(A–F)** VO2, VCO2 and RER in mice under resting conditions (*n* = 8); **(G–L)** VO2, VCO2 and RER in mice during exercise; **(M)** BMR in mice at 30 °C constant temperature (*n* = 8); **(N–P)** Grip strength, exhaustive swimming and exhaustive running results (*n* = 7); **(Q)** Mechanical work output in mice (*n* = 7). **(R–U)** 10-min swimming test (Lactate test, *n* = 6). Results are expressed as mean ± SEM. *p < 0.05, **p < 0.01, ***p < 0.001 compared to the Vehicle group.

Before gavage intervention, no significant differences were observed in the parameters related to energy metabolism and heat production among the mouse groups (Supplementary Figure S3). After 4 weeks of FSP intervention, the assessment revealed that, compared with the vehicle group, mice in all FSP groups exhibited significantly elevated oxygen consumption (VO2) and carbon dioxide exhalation (VCO2) under different states (Figures 3A–D, G–J). However, no significant differences in the respiratory exchange ratio (RER) were detected among groups (Figures 3E, F, K, L). Under environmental conditions of 30 °C, the basal metabolic rate (BMR) was increased in all FSP groups, with the most pronounced effect observed in the FSP-2X group (Figure 3M).

In terms of motor behavior, compared with the vehicle group, FSP-1X, FSP-1.5X, and FSP-2X groups significantly enhanced forelimb grip strength (Figure 3N) and prolonged exhaustive swimming time (Figure 3O). Additionally, the FSP-2X group demonstrated significant improvements in exhaustive running duration and mechanical work output (Figures 3P, Q).

Through a 10-min swimming test (Figures 3R–U), we observed that there were no significant differences in baseline blood lactate levels among the mouse groups before swimming. However, blood lactate levels significantly increased after exercise, particularly in the vehicle group, which showed the most pronounced rise. Calculations revealed that, compared with the vehicle group, the lactate production rate was notably reduced in the FSP-2X group, and the lactate clearance rate was significantly enhanced in the FSP groups, suggesting that FSP holds substantial potential in promoting lactate metabolism.

### 3.3 Effects of FSP on fatigue-associated biochemical markers after aerobic exercise

Table 1 summarizes the changes in multiple biochemical indicators in tissue, serum, and urine samples from four groups of mice after FSP intervention combined with aerobic exercise training. Regarding liver function indicators, FSP-1.5X and 2X groups significantly reduced serum AST and ALT concentrations. Elevated levels of these transaminases are commonly associated with various liver diseases. Meanwhile, all FSP groups showed a trend toward decreased serum LDH levels, though only the FSP-1X and 2X groups reached statistical significance, suggesting that FSP may regulate lactate metabolism. In metabolic parameters, all FSP groups significantly lowered serum total cholesterol (TC), indicating potential lipid metabolism modulation. Furthermore, the FSP-2X group exhibited significant reductions in creatine kinase (CK) and serum uric acid (UA), suggesting that this intervention may help alleviate muscle damage and improve purine metabolism.

**Table 1:** Effect of FSP on organ, serum and urine biochemical indices.

Parameter	Vehicle	FSP-1X	FSP-1.5X	FSP-2X	Trend analysis
Organ					
Hepatic glycogen (mg/g)	43.05 ± 6.807	43.59 ± 3.947	51.93 ± 6.358^*^	53.41 ± 5.589^*^	*p* = 0.0074^**^
Hepatic XOD (U/gprot)	21.87 ± 2.52	27.24 ± 6.78	23.81 ± 6.05	25.33 ± 8.38	*p* = 0.5944
Muscle glycogen (mg/g)	5.281 ± 0.6505	5.472 ± 0.4923	6.827 ± 1.527^*^	6.624 ± 0.5978^*^	*p* = 0.0152^*^
Serum					
AST (U/L)	105.9 ± 33.62	86.72 ± 27.97	63.45 ± 21.46^*^	68.60 ± 12.32^*^	*p* = 0.0336^*^
ALT (U/L)	56.58 ± 14.29	44.69 ± 10.67	37.20 ± 9.453^*^	33.57 ± 8.246^**^	*p* = 0.0080^**^
ALP (U/L)	4.855 ± 2.414	6.071 ± 1.302	5.598 ± 2.056	5.503 ± 1.976	*p* = 0.7658
LDH (U/L)	356.9 ± 76.39	226.7 ± 44.67^*^	239.0 ± 120.6	192.8 ± 88.69^*^	*p* = 0.0211^*^
CK (U/L)	312.3 ± 26.67	235.2 ± 103.1	272.0 ± 42.96	196.5 ± 58.20^*^	*p* = 0.0326^*^
TP (μg/ml)	801.6 ± 139.9	974.6 ± 90.32	1,019 ± 220.1	834.7 ± 106.2	*p* = 0.0768
TC (mmol/L)	3.893 ± 0.6458	2.772 ± 0.3357^**^	3.057 ± 0.4530^*^	3.047 ± 0.6310^*^	*p* = 0.0096^**^
TG (mmol/L)	1.336 ± 0.3068	1.054 ± 0.2541	1.167 ± 0.2554	1.206 ± 0.3688	*p* = 0.4590
Glucose (mmol/L)	11.12 ± 2.872	9.111 ± 3.648	11.26 ± 3.966	11.03 ± 3.106	*p* = 0.6665
Creatinine (μmol/L)	14.26 ± 2.912	9.463 ± 3.643	12.11 ± 5.106	12.47 ± 3.707	*p* = 0.2894
BUN (mmol/L)	6.713 ± 1.615	5.766 ± 0.9245	6.152 ± 0.8777	6.046 ± 0.9014	*p* = 0.5343
UA (μmol/L)	23.28 ± 7.661	16.72 ± 4.644	18.59 ± 5.807	11.45 ± 11.07^*^	*p* = 0.0957
Ammonia (mmol/L)	9.078 ± 2.312	9.658 ± 1.646	9.658 ± 2.466	8.185 ± 1.146	*p* = 0.6389
Urine					
Creatinine (μmol/L)	354.2 ± 110.72	362.6 ± 118.14	321.8 ± 84.01	375.0 ± 35.12	*p* = 0.7837
UA (μmol/L)	9.009 ± 3.046	9.223 ± 3.854	8.687 ± 4.244	6.649 ± 4.124	*p* = 0.6428
Na^+^ (mmol/L)	90.10 ± 9.518	87.67 ± 12.76	93.41 ± 13.40	92.59 ± 22.31	*p* = 0.8977
K^+^ (mmol/L)	3.265 ± 0.5955	3.930 ± 0.9727	3.501 ± 0.7348	3.647 ± 0.4218	*p* = 0.4147

^*^p < 0.05, ^**^p < 0.01, ^***^p < 0.001 compared to the vehicle group.

As critical energy reserves, hepatic and muscle glycogen levels are closely linked to exercise endurance. This study further demonstrated that FSP intervention significantly increased glycogen storage in mouse liver and muscle, with the most pronounced effects observed in the FSP-1.5X and 2X groups, providing a potential mechanistic basis for the enhanced exercise endurance phenotype.

### 3.4 Analysis and validation of liver RNA-Seq results

**Figure 4:**
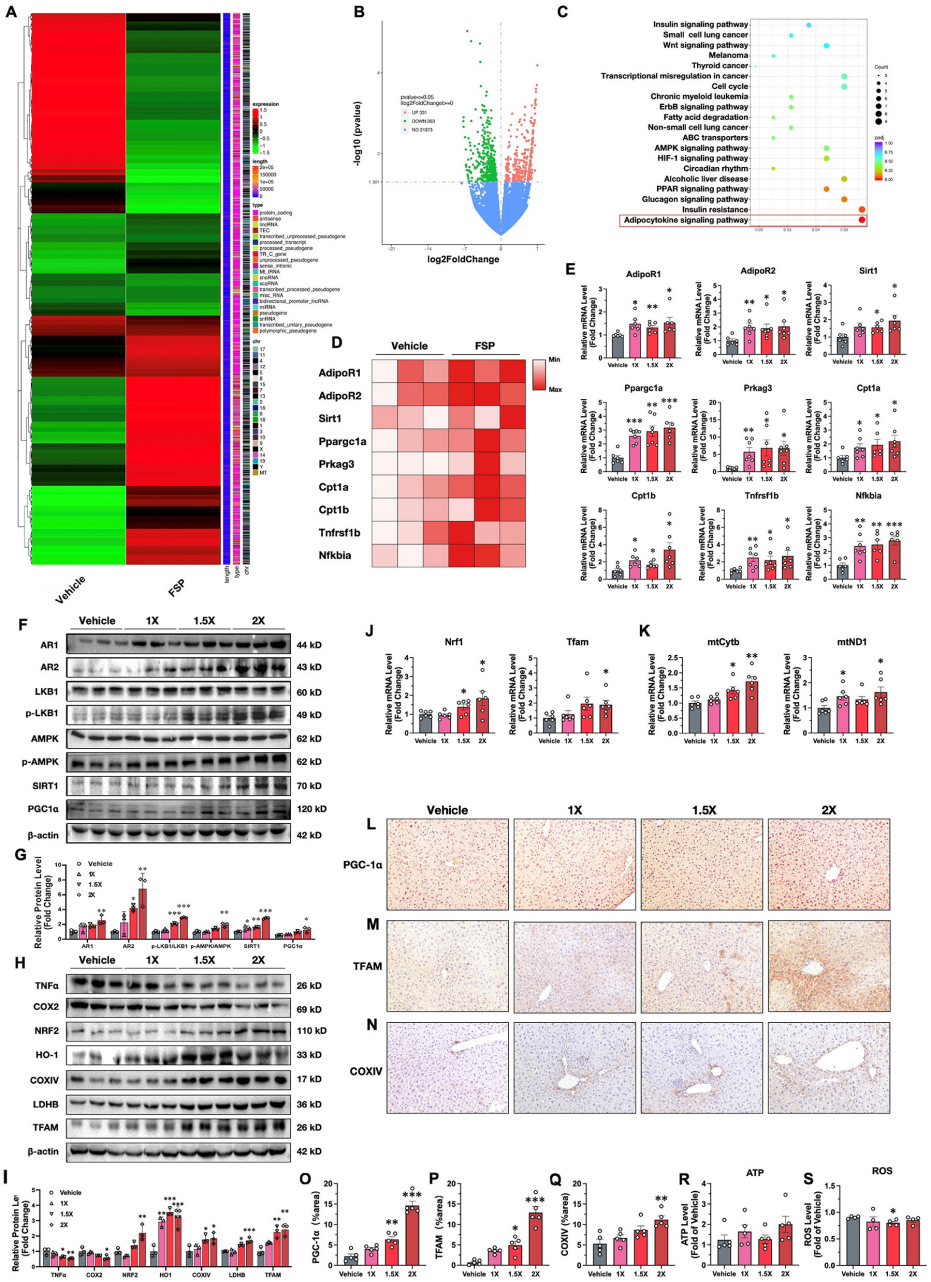
RNA-Seq analysis and validation of mouse liver following FSP intervention. **(A)** Heatmap of hepatic gene expression profiles in vehicle group and FSP-2X group, with red and green indicating up- and down-regulated genes respectively (*n* = 3); **(B)** Volcano plot displaying overall distribution of DEGs in FSP-treated liver tissues (*n* = 3); **(C)** Bubble chart of KEGG pathway enrichment analysis for DEGs (*n* = 3); **(D)** Heatmap visualization of differentially expressed genes (*n* = 3); **(E)** mRNA expression analysis of target genes (*n* = 6); **(F, G)** Expression levels and semi-quantitative analysis of key proteins in AdipoRs-AMPK signaling pathway (*n* = 3); **(H, I)** Regulatory effects of FSP on proteins related to inflammation, oxidative stress, mitochondrial function and lactate metabolism (*n* = 3); **(J)** mRNA levels of mitochondrial biogenesis genes Nrf1 (NRF1) and Tfam (TFAM) (*n* = 6); **(K)** Mitochondrial DNA copy number in mouse liver tissues (*n* = 6); **(L–Q)** Immunohistochemical staining and statistical analysis of PGC1α, TFAM and COXIV proteins (Scale bar: 20 μm, Total magnification: 200x, *n* = 5); **(R)** Hepatic ATP content (*n* = 5); **(S)** ROS levels in liver tissues (*n* = 4). Results are expressed as mean ± SEM. ^*^p < 0.05, ^**^p < 0.01, ^***^p < 0.001 compared to the vehicle group.

To further investigate the mechanism by which FSP improves exercise capacity, we performed transcriptome sequencing on liver tissues from vehicle (*n* = 3) and FSP-2X (*n* = 3) groups. Heatmap analysis (Figure 4A) revealed clear clustering and distinct intergroup separation, indicating widespread gene expression differences. A total of 331 upregulated and 363 downregulated genes were identified (Figure 4B). KEGG pathway enrichment analysis (Figure 4C) suggested the adipocytokine signaling pathway may play a key role in FSP's biological effects. Differential gene expression analysis (Figure 4D) further indicated this process may be closely associated with activation of the adiponectin receptor (AdipoRs)-mediated AMPK signaling pathway.

Subsequent mRNA-level validation of key genes (Figure 4E) demonstrated that all FSP-treated groups significantly upregulated AdipoR1 and AdipoR2 expression, while concurrently enhancing transcription of Prkag3, a crucial AMPK subunit. The cellular energy sensor SIRT1 showed significantly increased expression in FSP-1.5X and 2X groups, accompanied by upregulation of its downstream target Ppargc1a (PGC1α). Furthermore, key genes involved in mitochondrial fatty acid β-oxidation (Cpt1a and Cpt1b) exhibited marked upregulation across all FSP-treated groups. Experimental results also indicated FSP intervention effectively promoted mRNA expression of several anti-inflammatory genes including Tnfrsf1b and Nfkibia, suggesting its potential role in modulating inflammatory responses.

Western blot analysis (Figures 4F, G) demonstrated that, compared with the vehicle group, FSP intervention dose-dependently up-regulated the protein expression levels of AdipoRs and their downstream key signaling molecules, including p-LKB1/LKB1, p-AMPK/AMPK, SIRT1, and PGC1α. This effect was particularly pronounced in the FSP-1.5X and/or 2X groups. Regarding oxidative stress and inflammatory regulation, FSP treatment significantly enhanced the expression of the antioxidant proteins NRF2 (FSP-2X group) and HO-1 (FSP-1X, 1.5X, and 2X groups), while effectively suppressing the expression of the pro-inflammatory proteins TNFα (FSP-1.5X and 2X groups) and COX2 (FSP-2X group). Furthermore, LDHB, a key enzyme in lactate metabolism regulated by PGC1α, showed significant up-regulation at the protein level (Figures 4H, I), providing a molecular explanation for the potential mechanism by which FSP promotes lactate clearance and metabolic conversion.

To further evaluate the impact of AdipoRs-AMPK axis activation on mitochondrial function, this study systematically assessed multiple mitochondrial function-related indicators. Results revealed that the mitochondrial function proteins COXIV and TFAM were significantly elevated in the FSP-2X group (Figures 4H, I). The mRNA expression of mitochondrial biogenesis-related genes NRF1 and TFAM also showed an increasing trend, reaching statistical significance in the FSP-2X group (Figure 4J). Concurrently, mitochondrial DNA (mtDNA) copy number in the liver tissues of this group was markedly increased (Figure 4K). As the central organelles for intracellular energy metabolism, mitochondrial functional status can be indirectly reflected by ATP and reactive oxygen species (ROS) levels. Experimental data indicated that FSP intervention exhibited a trend toward increased ATP production (Figure 4R), and the FSP-1.5X group effectively reduced ROS levels (Figure 4S). Immunohistochemical analysis further confirmed that the expression of mitochondrial-related proteins such as PGC1α, TFAM, and COXIV progressively increased with higher FSP doses (Figures 4L–Q), collectively indicating that FSP has a definite positive effect in promoting mitochondrial biogenesis and functional improvement.

### 3.5 FSP serum activates AdipoRs-AMPK pathway and improves mitochondrial function in HepG2 cells

**Figure 5:**
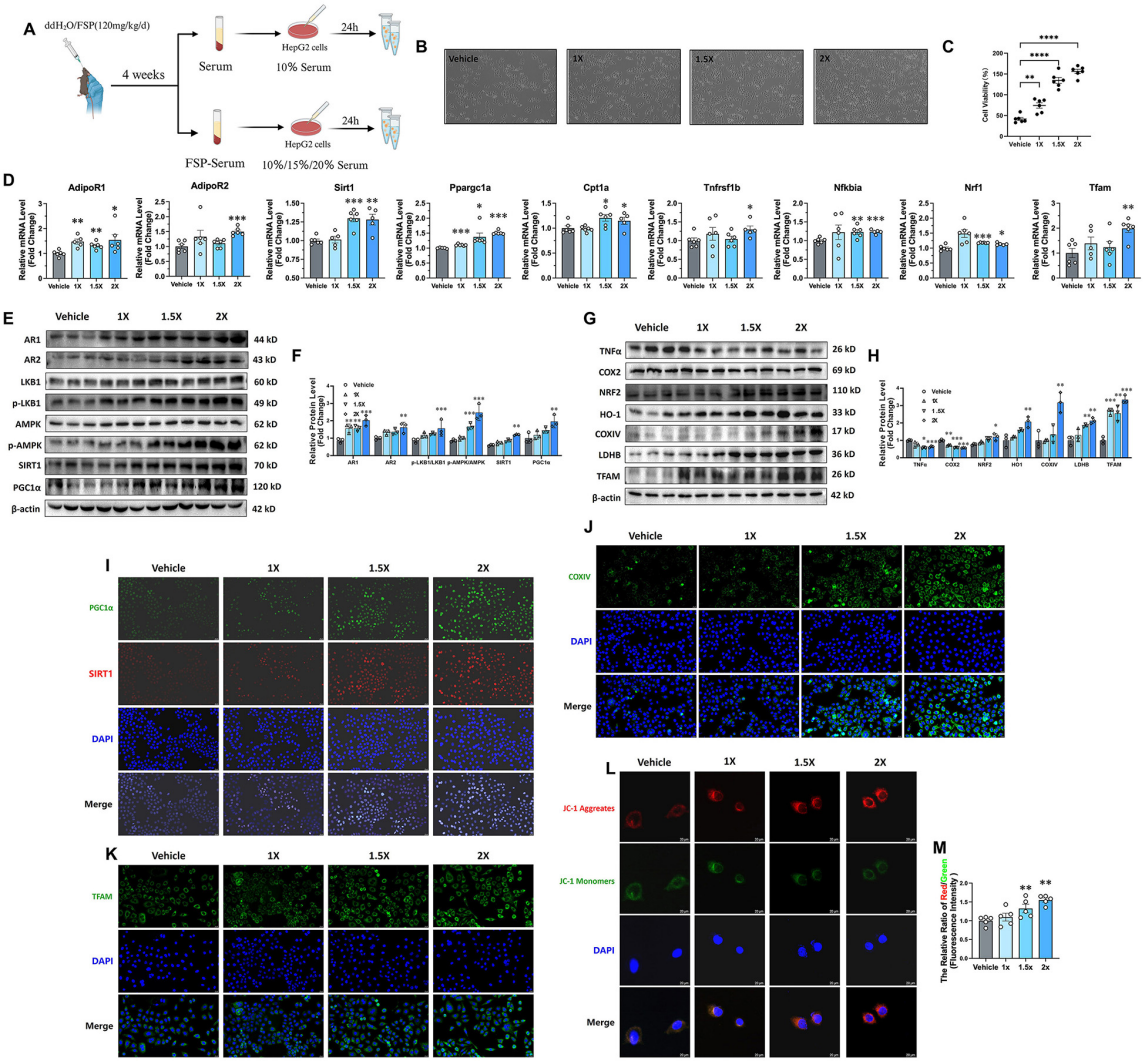
Effects of FSP serum treatment on gene expression in HepG2 cells. **(A)** Experimental design of FSP serum treatment in HepG2 cells (Created in https://BioRender.com); **(B, C)** Impact of FSP serum on HepG2 cell proliferation capacity and quantitative analysis (*n* = 6); **(D)** Transcript levels of differentially expressed genes and mitochondrial biogenesis-related genes (Nrf1/NRF1, Tfam/TFAM) in HepG2 cells (*n* = 6); **(E, F)** Protein expression and semi-quantification of key AdipoRs-AMPK pathway components in HepG2 cells (*n* = 3); **(G, H)** Effects of FSP serum on inflammation, oxidative stress, mitochondrial function and lactate metabolism-related proteins with semi-quantitative analysis (*n* = 3); **(I)** Immunofluorescence co-localization of SIRT1 and PGC-1α proteins in HepG2 cells (Scale bar: 20 μm, Total magnification: 200x); **(J, K)** Immunofluorescence detection of mitochondrial function proteins COXIV and TFAM (Scale bar: 20 μm, Total magnification: 200x); **(L, M)** Mitochondrial membrane potential assay (JC-1) and analysis (Scale bar: 20 μm, Total magnification: 200x, *n* = 5). Results are expressed as mean ± SEM. *p < 0.05, **p < 0.01, ***p < 0.001 compared to the vehicle group.

This study employed a medicated serum approach by supplementing HepG2 cell cultures with FSP-containing serum at varying concentrations (Figure 5A) to systematically evaluate its cellular effects. Microscopic observation revealed no significant morphological abnormalities in any FSP serum-treated groups compared to controls, while cell numbers were substantially increased, indicating no apparent cytotoxicity and potential proliferative effects of FSP serum (Figures 5B, C).

At the molecular level, Western blot analysis demonstrated dose-dependent upregulation of multiple core components within the AdipoRs-AMPK signaling pathway (p-LKB1/LKB1, p-AMPK/AMPK, SIRT1, PGC1α), with statistically significant alterations particularly evident in the FSP-20% serum group (Figures 5E, F). RT-qPCR results further confirmed this trend at the transcriptional level, showing marked upregulation of mRNA expression for genes including AdipoR1, AdipoR2, SIRT1, Ppargc1a, Tnfrsf1b, and Nfkibia in the FSP-20% serum group (Figure 5D). Additionally, LDHB, a key enzyme in lactate metabolism, exhibited significantly enhanced protein expression in the FSP-15% and 20% serum groups (Figures 5G, H). Regarding oxidative stress and inflammatory regulation, FSP serum markedly promoted the expression of antioxidant proteins NRF2 and HO-1 while effectively suppressing pro-inflammatory proteins TNFα and COX2 (Figures 5G, H).

At the cellular level, FSP serum similarly demonstrated positive regulatory effects on mitochondrial function. RT-qPCR analysis revealed significantly enhanced mRNA expression of mitochondrial biogenesis genes NRF1 and TFAM in the FSP-20% serum group (Figure 5D). Western blot analysis further confirmed concurrent elevation of mitochondrial function-related proteins TFAM and COXIV (Figures 5G, H). Immunofluorescence staining showed substantially increased fluorescence intensity of mitochondrial-associated proteins SIRT1 & PGC1α, TFAM, and COXIV under FSP-20% serum conditions (Figures 5I–K). As a crucial indicator of mitochondrial functional status, JC-1 staining demonstrated elevated red/green fluorescence intensity ratios across all FSP serum treatments, with the most pronounced effects in FSP-15% and 20% serum groups (Figures 5L, M). These consistent findings collectively indicate that FSP serum effectively activates the AdipoRs-AMPK signaling pathway at the cellular level, thereby promoting mitochondrial biogenesis, improving mitochondrial function, and exerting synergistic anti-inflammatory and antioxidant effects.

### 3.6 AMPK inhibition reverses the enhancing effects of FSP serum on mitochondrial function and lactate metabolism in HepG2 cells

**Figure 6:**
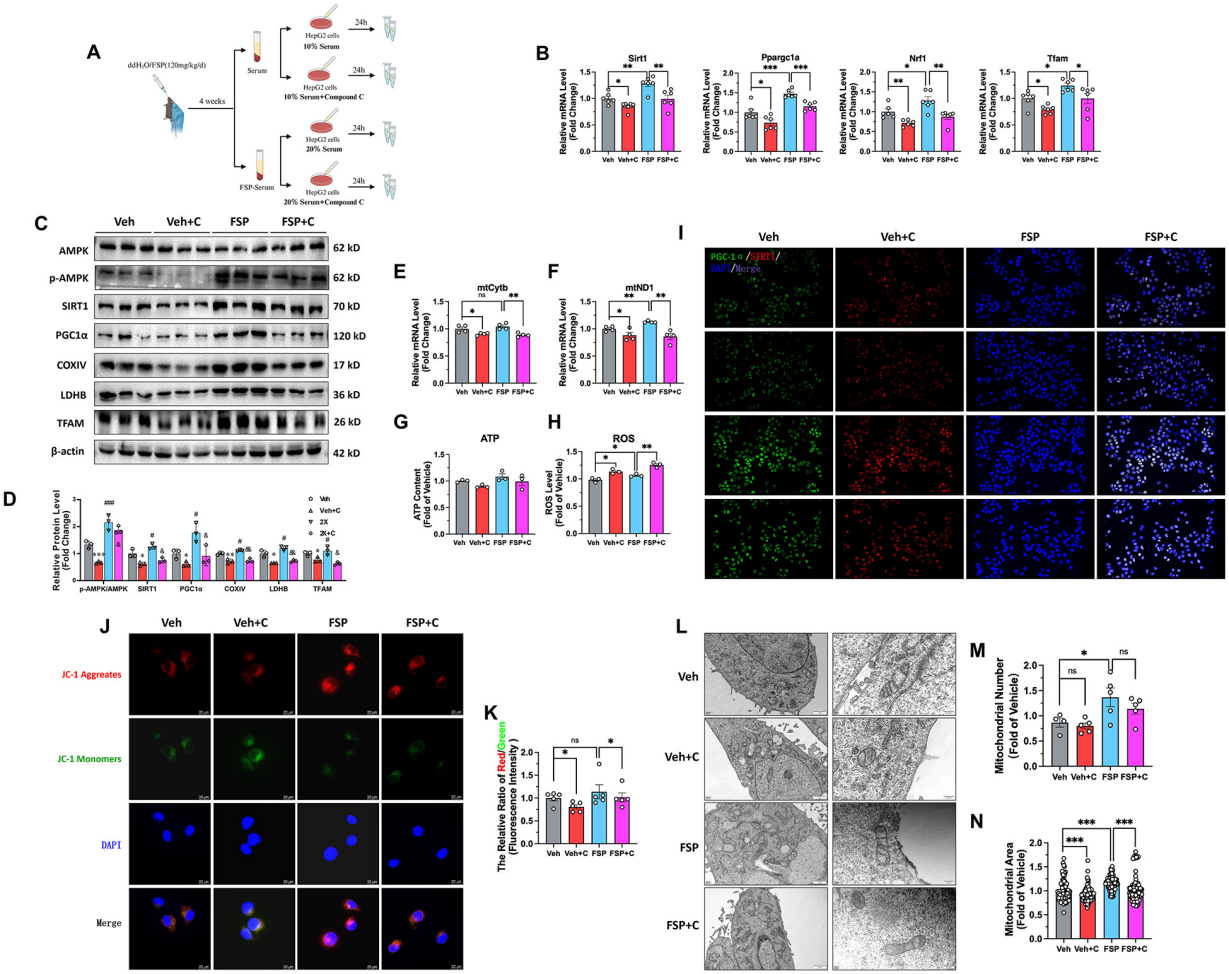
AMPK inhibition reverses biological effects of FSP serum intervention. **(A)** AMPK inhibition experiment in HepG2 cells under FSP serum intervention (Created in https://BioRender.com); **(B)** Transcript levels of AMPK downstream effectors (SIRT1, PGC-1α) and mitochondrial genes (Nrf1/NRF1, Tfam/TFAM) (*n* = 6); **(C, D)** Protein expression and semi-quantitative analysis of AMPK pathway molecules after AMPK inhibitor intervention (*n* = 3); **(E, F)** Mitochondrial DNA copy number analysis (*n* = 4); **(G)** Measurement of cellular ATP content (*n* = 3); **(H)** Detection of cellular reactive oxygen species (ROS) levels (*n* = 3); **(I)** SIRT1 and PGC-1α immunofluorescence co-localization (Scale bar: 20 μm, Total magnification: 200x); **(J, K)** Mitochondrial membrane potential (JC-1) staining and analysis (Scale bar: 20 μm, Total magnification: 200x, *n* = 5); **(L–N)** TEM analysis of mitochondrial ultrastructure with mitochondrial number (*n* = 5) and area quantification (*n* = 100) (Scale bar: 1/0.2 μm, Total magnification: 20,000/80,000x). Results are expressed as mean ± SEM. *p < 0.05, **p < 0.01, ***p < 0.001compared to the vehicle gro,Q22up.

Multiple signal transduction pathways exist downstream of adiponectin receptors. To clarify whether FSP exerts its effects through the AdipoRs-AMPK signaling pathway, this study introduced a specific AMPK inhibitor (Compound C) into both vehicle serum and high-concentration FSP serum treatment groups (Figure 6A) for pathway dependency verification. Protein and gene expression analyses revealed that upon AMPK inhibition, both transcriptional and translational levels of SIRT1 and PGC1α were significantly downregulated (Figures 6C, D). Concurrent with the suppression of these upstream signaling molecules, expression of downstream mitochondrial function protein COXIV and key biogenesis factors NRF1 and TFAM were also reduced (Figures 6B–D), accompanied by decreased mitochondrial DNA (mtDNA) copy number (Figures 6E, F), diminished ATP production (Figure 6G), and a significant rebound in ROS levels (Figure 6H). Mitochondrial membrane potential assessment further showed a marked decline in the red/green fluorescence intensity ratio following inhibitor intervention (Figures 6J, K).

Ultrastructural observation via transmission electron microscopy (Figures 6L–N) indicated that upon AMPK inhibition, mitochondrial numbers tended to decrease, cross-sectional areas were significantly reduced, and cristae density was diminished, suggesting AMPK plays a critical role in FSP-mediated maintenance of mitochondrial morphology and function. Immunofluorescence staining further corroborated this conclusion: fluorescence intensities of SIRT1 & PGC1α (Figure 6I) as well as TFAM and COXIV (Supplementary Figure S4) were substantially attenuated after inhibitor treatment. Moreover, expression of LDHB, a key enzyme in lactate metabolism, was notably reduced upon AMPK inhibition (Figures 6C, D). These consistent results demonstrate that the mitochondrial function enhancement and lactate metabolism regulation mediated by FSP at the cellular level are specifically dependent on activation of the AMPK signaling pathway.

### 3.7 Screening of key active components in FSP: taxifolin and apigenin

The aforementioned findings suggest that FSP may exert its anti-fatigue and exercise-enhancing effects by activating the AdipoRs-AMPK signaling pathway, thereby enhancing mitochondrial function and improving energy metabolism. To systematically identify the key active components in FSP responsible for mediating this process, we established a comprehensive multi-index evaluation system (Table 2) integrating molecular docking simulations, oral bioavailability (data source: https://www.tcmsp-e.com/), drug-likeness (data source: http://tcmspw.com/), and intestinal absorption efficiency (data source: http://www.swissadme.ch/).

**Table 2:** Systematic screening of FSP active ingredients.

No.	Compounds	Classes	Formula	Molecular weight (MW; g/mol)	Docking score with Adipor1 (ΔG, kcal/mol)	Docking score with Adipor2 (ΔG, kcal/mol)	Oral bioavailability (OB, %)	Drug-likeness (DL)	Gastrointestinal absorption	OB normalization	Normalization of docking scores against AdipoR1	Normalization of docking scores against AdipoR2	Weighted algorithm (35% AdipoR1 + 35%AdipoR2 + 30% OB)	Ranking
*Smilax glabra* Roxb.														
1	Astilbin (main ingredient)	Flavonoid	C_21_H_22_O_11_	450.43	−8.9	−7.5	36.46	0.74	Low	0.61261098	0.48	0	0.351783294	7
2	Isoastilbin	Flavonoid	C_21_H_22_O_11_	450.43	−8.4	−8.9	27.05	0.74	Low	0.442109078	0.28	1	0.580632723	3
3	Neoastilbin	Flavonoid	C_21_H_22_O_11_	450.43	−9	−8.2	40.54	0.74	Low	0.686537416	0.52	0.5	0.562961225	4
4	Neoisoastilbin	Flavonoid	C_21_H_22_O_11_	450.43	−7.7	−7.8	36.46	0.74	Low	0.61261098	0	0.214285714	0.258783294	8
5	Resveratrol	Polyphenol	C_14_H_12_O_3_	228.26	−9	−8	19.07	0.11	High	0.297517666	0.52	0.357142857	0.3962553	6
6	Taxifolin	Flavonoid	C_15_H_12_O_7_	304.27	−10.2	−8.3	57.84	0.27	High	1	1	0.571428571	0.85	1
7	Engeletin	Flavonoid	C_21_H_22_O_10_	434.43	−9.1	−8.4	2.65	0.7	Low	0	0.56	0.642857143	0.421	5
8	Epicatechin	Flavonoid	C_15_H_14_O_6_	290.29	−9.8	−8.3	28.93	0.24	High	0.47617322	0.84	0.571428571	0.636851966	2
*Ficus hirta* Vahl.														
9	Apigenin (quality marker)	Flavonoid	C_15_H_10_O_5_	270.25	−10.2	−8.3	23.06	0.21	High	0.510980592	0.771428571	0.76	0.689294178	3
10	Psoralen (quality marker)	Coumarin	C_11_H_6_O_3_	186.17	−8.6	−6.8	33.06	0.1	High	0.766343207	0.314285714	0.16	0.395902962	6
11	Bergapten (quality marker)	Coumarin	C_12_H_8_O_4_	216.2	−8.5	−7.1	42.21	0.13	High	1	0.285714286	0.28	0.498	4
12	Luteolin	Flavonoid	C_15_H_10_O_6_	286.25	−10.4	−8.5	36.16	0.25	High	0.845505618	0.828571429	0.84	0.837651685	1
13	Vitexin	Flavonoid	C_21_H_20_O_10_	432.41	−8.9	−7.6	3.05	0.71	Low	0	0.4	0.48	0.308	8
14	Curdione	Sesquiterpene	C_15_H_24_O_2_	236.39	−8.0	−6.4	38.94	0.08	High	0.916496425	0.142857143	0	0.324948927	7
15	Naringin	Flavonoid	C_15_H_12_O_5_	580.59	−11.0	−8.9	6.92	0.78	Low	0.098825332	1	1	0.7296476	2
16	7-hydroxycoumarin	Coumarin	C_9_H_6_O_3_	162.15	−7.5	−7.9	27.37	0.05	High	0.621041879	0	0.6	0.396312564	5

First, we compiled the major compounds quantitatively reported via liquid chromatography or mass spectrometry in *Smilax glabra* Roxb. (23–25) and *Ficus hirta* Vahl. (9, 26, 27), selecting the top eight most frequently reported components from each herb. These 16 compounds were then subjected to molecular docking analysis with AdipoR1 and AdipoR2 (Supplementary Figures S5, S6) and ranked using a weighted scoring method. Scoring criteria included the lowest binding free energy to AdipoR1 (weight: 35%) and AdipoR2 (weight: 35%), along with oral bioavailability (weight: 30%). Based on the scores, the top three candidate components from each herb were selected.

Subsequently, a comprehensive evaluation was performed considering drug-likeness (>0.18), intestinal absorption efficiency (high/low), and actual content in the raw herbs. Results indicated that taxifolin from *Smilax glabra* Roxb. performed exceptionally well across multiple parameters. In *Ficus hirta* Vahl., although luteolin ranked highest overall, its low content and comparable evaluation parameters to apigenin—a known quality marker for this herb—led to the preferential selection of the more abundant apigenin as the representative component. Naringenin, ranked second, was excluded from further validation due to its low absorption efficiency and limited content. In summary, through multiple rounds of screening and comprehensive evaluation, taxifolin and apigenin were ultimately identified as the primary candidate active components for subsequent experimental validation.

### 3.8 Validation of targeted interaction between taxifolin/apigenin and AdipoRs

**Figure 7:**
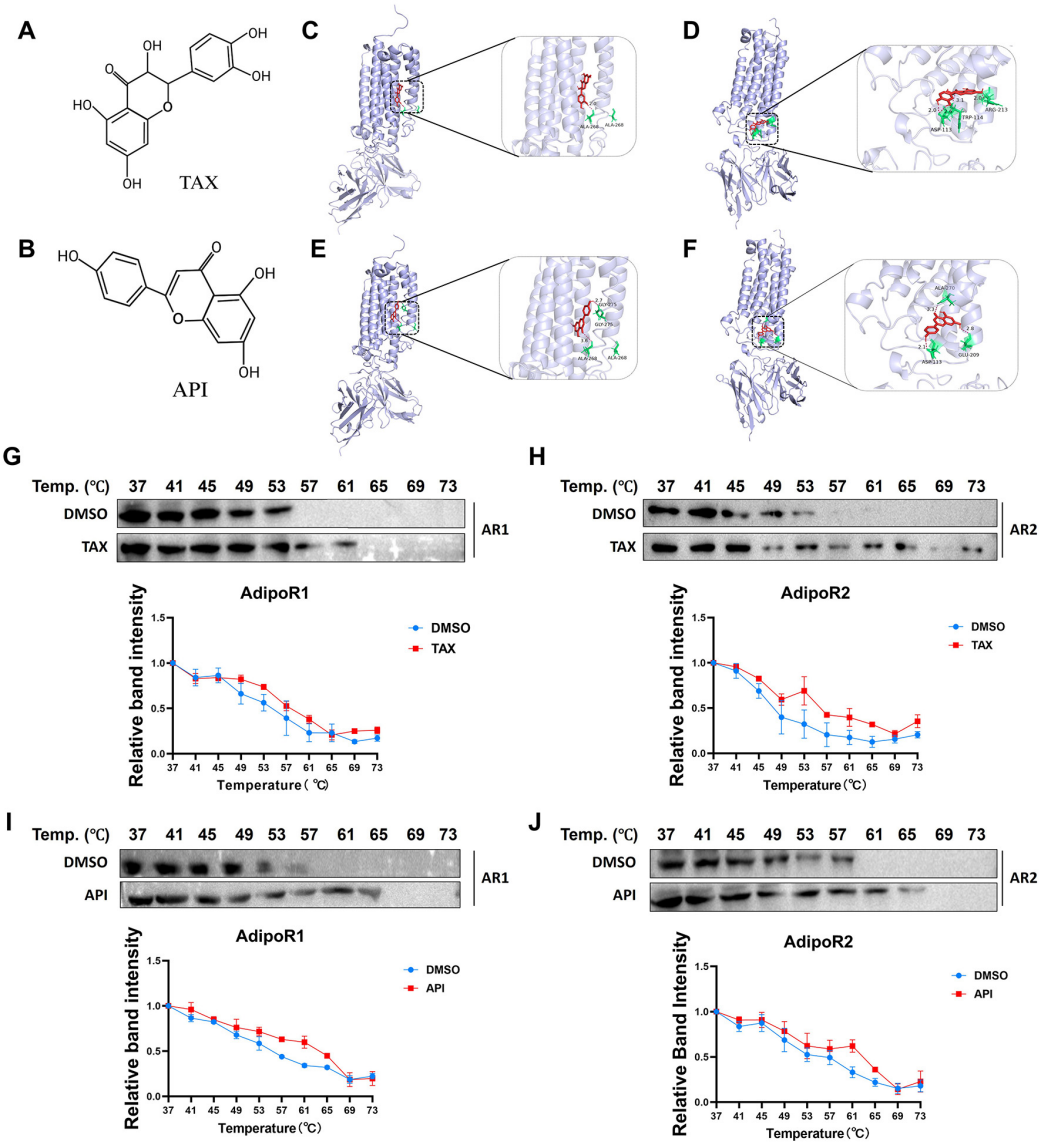
Validation of targeted effects of taxifolin and apigenin on AdipoRs. **(A, B)** Chemical structures of taxifolin and apigenin (Created in https://BioRender.com); **(C, D)** Molecular docking analysis of taxifolin with AdipoRs using AutoDock; **(E, F)** Molecular docking analysis of apigenin with AdipoRs using AutoDock; **(G, H)** CETSA-WB assay validating thermal stability of taxifolin-AdipoRs interaction (*n* = 3); **(G, H)** CETSA-WB experiments to verify thermal stability of taxifolin and AdipoRs (*n* = 3); **(I, J)** CETSA-WB experiments to verify thermal stability of apigenin and AdipoRs (*n* = 3).

Figure 7A shows the chemical structure of taxifolin, and Figure 7B shows the chemical structure of apigenin. Molecular docking analysis results (Figures 7C–F) indicate that the binding free energy of both taxifolin and apigenin to AdipoR2 was −8.2 kcal/mol, while the binding free energy to AdipoR1 was as low as −10.2 kcal/mol, suggesting that both compounds have the potential to bind to AdipoRs and exhibit higher affinity for AdipoR1. To validate these computational results, this study further employed the Cellular Thermal Shift Assay (CETSA), based on the principle of thermal stability changes after ligand-target protein binding, to experimentally verify the interaction between the compounds and AdipoRs.

Results (Figures 7G–J) demonstrated that, compared to the vehicle group, the thermal stability of AdipoRs in the supernatant of drug-treated groups was significantly enhanced, confirming the reliability of the molecular docking predictions. Under conditions where 50% of the target protein underwent thermal denaturation, the denaturation temperature of AdipoR1 in the treatment groups was higher than that of AdipoR2, further suggesting a more stable binding of both compounds to AdipoR1. This experimental outcome aligns with the conclusions drawn from the molecular docking calculations.

### 3.9 Improving effects of taxifolin and/or apigenin on energy metabolism and exercise performance in mice

The experimental protocol for monomer compounds was established with reference to previous FSP whole-animal study procedures, with specific design details illustrated in Figure 2A. Throughout the experimental period, no significant abnormalities were observed in the administered groups regarding gross morphology (Figure 2B), body weight progression (Figure 2C), weights of major organs, or adipose tissue proportions (Supplementary Table S3). Basic physiological parameters monitored via metabolic caging systems, including food and water intake, also showed no notable intergroup differences (Supplementary Figures S7A–E), indicating that taxifolin and apigenin, at the administered doses, produced no observable adverse effects on the fundamental physiological status of the mice.

In terms of energy metabolism (Figures 2D–O), combined intervention with taxifolin and apigenin significantly enhanced oxygen consumption (VO2) and carbon dioxide production (VCO2) in mice under both resting and exercising conditions, improved overall energy conversion efficiency, and effectively increased the basal metabolic rate (BMR) under high-temperature conditions at 30 °C (Figure 2P). Regarding motor function (Figures 2Q–T), both compounds significantly enhanced forelimb grip strength, prolonged exhaustive swimming and running durations, increased mechanical work output, and promoted post-exercise blood lactate clearance (Figures 2U–X). Notably, although both taxifolin and apigenin alone exerted certain beneficial effects, apigenin demonstrated stronger efficacy across multiple parameters. Moreover, the combined administration group exhibited significant synergistic enhancement in most outcomes, suggesting complementary or synergistic interactions between the two compounds in regulating energy metabolism and exercise endurance.

Biochemical analysis of blood, urine, and tissue samples from mice treated with monomer compounds (Supplementary Table S4) revealed that both taxifolin and apigenin significantly reduced serum lactate dehydrogenase (LDH) levels compared to the vehicle group, with the combination group showing the most pronounced reduction. Regarding creatine kinase (CK), both taxifolin alone and its combination with apigenin significantly decreased CK levels, suggesting their potential in alleviating exercise-induced muscle damage.

Further observations indicated that serum glucose concentration was markedly reduced in the taxifolin alone and combination groups, implying a potential role in regulating glucose homeostasis. Notably, the combined intervention significantly enhanced glycogen storage in both liver and muscle tissues, providing crucial evidence for explaining the synergistic effect on improved exercise endurance.

### 3.10 Synergistic activation of AdipoRs-AMPK pathway by taxifolin and apigenin enhances mitochondrial function in HepG2 cells

**Figure 8:**
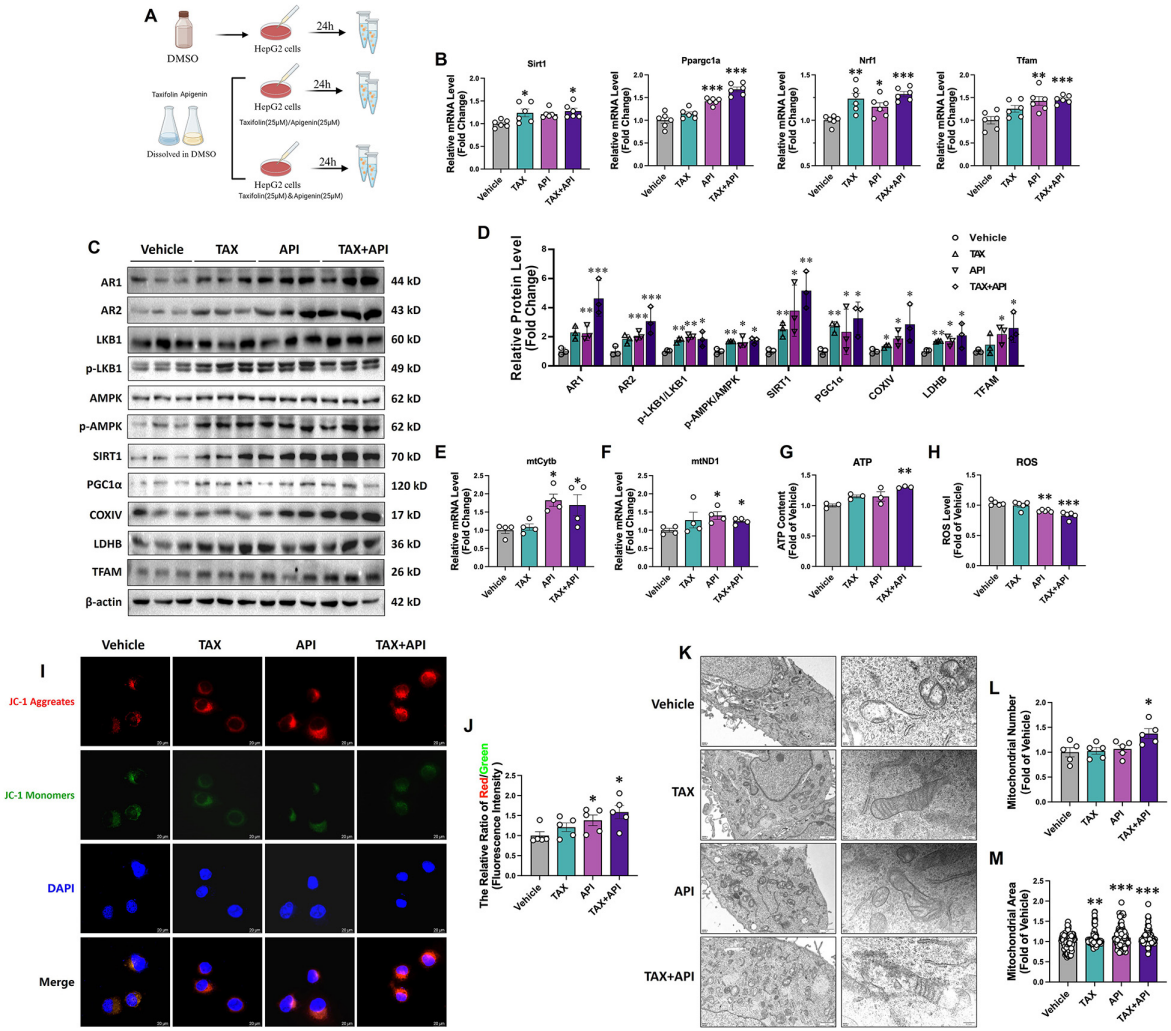
Synergistic activation of the AdipoRs-AMPK pathway by taxifolin and apigenin combination **(A)** Schematic design of monomer compound cell experiments (Created in https://BioRender.com); **(B)** mRNA expression levels of STRT1, Ppargc1a, Nrf1 (NRF1), and Tfam (TFAM) (*n* = 6); **(C, D)** Protein expression in AdipoRs-AMPK pathway and mitochondrial function (*n* = 3); **(E, F)** Mitochondrial DNA copy number detection (*n* = 6); **(G)** Intracellular ATP content measurement (*n* = 3); **(H)** Detection of reactive oxygen species (ROS) levels (*n* = 5); **(I, J)** Measurement of mitochondrial membrane potential (JC-1) (Scale bar: 20 μm, Total magnification: 200x, *n* = 5); **(K–M)** Transmission electron microscopy (TEM) observation of mitochondrial ultrastructure (Scale bar: 1/0.2 μm, Total magnification: 20,000/80,000x), statistical analysis of mitochondrial quantity (*n* = 5) and mitochondrial area (*n* = 100). Results are expressed as mean ± SEM. *p < 0.05, **p < 0.01, ***p < 0.001 compared to the vehicle group.

The experimental procedure for monomer compounds in cell culture is shown in Figure 8A. Western blot analysis demonstrated that both taxifolin and apigenin significantly upregulated the expression of key proteins in the AdipoRs-AMPK signaling pathway (Figures 8C, D). Moreover, the combined intervention exhibited superior activation effects on most targets compared to individual treatments, suggesting a synergistic interaction.

Regarding mitochondrial function, the combination group showed multi-dimensional improvements: transcription levels of mitochondrial biogenesis-related genes NRF1 and TFAM were significantly increased (Figure 8B), mtDNA copy number was markedly elevated (Figures 8E, F), ATP production was significantly increased (Figure 8G), ROS levels were effectively reduced (Figure 8H), and mitochondrial membrane potential was substantially enhanced (Figures 8I, J). Ultrastructural observation by transmission electron microscopy further revealed that combined intervention promoted mitochondrial morphological remodeling, characterized by increased cross-sectional area, greater mitochondrial number, and enhanced cristae density (Figures 8K–M), thereby validating synergistic enhancement of mitochondrial function at both molecular and morphological levels.

Additionally, the combined intervention significantly up-regulated the expression of antioxidant protein NRF2 and its downstream target HO-1, while simultaneously suppressing protein levels of pro-inflammatory factors TNFα and COX2. At the gene expression level, multiple key genes including AdipoR1, AdipoR2, Prkag3, Cpt1a, Tnfrsf1b, and Nfkibia were significantly upregulated in the combination group (Supplementary Figure S9). These cellular-level results are highly consistent with the previously observed pharmacological effects of the FSP complex in animal models, systematically validating taxifolin and apigenin as the core active components in FSP responsible for regulating energy metabolism and mitochondrial function.

## 4 Discussion

Physical fatigue is one of the core factors constraining exercise performance (28). With the in-depth development of national fitness campaigns, nutritional recovery strategies following high-intensity training have become a focal point in the field of sports medicine (29). Short-term high-intensity training can induce microdamage to muscle fibers and trigger inflammatory responses, leading to delayed onset muscle soreness and fatigue accumulation, which subsequently reduce training efficacy (30). Conversely, medium-to-long-term, moderate-to-high-intensity training results in sustained accumulation of intermediate metabolites such as lactate, exacerbating muscle fatigue and impairing exercise capacity (31). In recent years, various traditional Chinese medicines (TCMs) and their extracts have demonstrated significant potential in enhancing exercise endurance (32, 33). The *Smilax glabra* Roxb. and *Ficus hirta* Vahl. (FSP), as a traditional herbal combination, has not yet been systematically elucidated regarding its mechanisms for improving exercise performance. Therefore, this study first established a mouse gavage model and confirmed that the three doses of FSP did not produce significant toxic or side effects under experimental conditions. Subsequent exercise behavioral experiments demonstrated that FSP significantly enhanced forelimb grip strength and prolonged exhaustive exercise time in mice. Further energy metabolism monitoring revealed that FSP intervention increased oxygen consumption (VO2) and carbon dioxide production (VCO2) in mice during both resting and exercising states, suggesting a potential mechanism for improving exercise performance through optimized energy metabolism efficiency. Additionally, serum and urinary biochemical analyses indicated that FSP significantly improved liver function markers (ALT and AST) and effectively reduced the accumulation of metabolic products such as TC, blood ammonia, and UA. Moreover, we found that FSP decreased serum concentrations of LDH and CK, which serve as specific markers for pyruvate-to-lactate conversion under hypoxic conditions (34) and muscle damage, respectively (35). These findings provide new experimental evidence clarifying the mechanism by which FSP alleviates fatigue in mice. Notably, medium and high concentrations of FSP (FSP-1.5X and 2X groups) significantly enhanced glycogen storage in mouse liver and muscle tissues. Adequate hepatic and muscular glycogen stores can effectively prolong endurance during high-intensity exercise and delay the onset of fatigue (36), which may represent another crucial mechanism underlying the anti-fatigue effects of FSP.

Based on hepatic transcriptome sequencing (RNA-Seq) results from preliminary animal experiments, this study reveals the core mechanism by which FSP enhances exercise performance: activation of the AMPK signaling pathway via AdipoRs (AdipoR1/AdipoR2). Previous studies have shown that AdipoRs promote nucleocytoplasmic translocation of liver kinase B1 (LKB1), thereby initiating AMPK phosphorylation. Activated AMPK not only directly activates peroxisome proliferator-activated receptor gamma coactivator 1-alpha (PGC-1α) (37), but also dynamically senses mitochondrial functional status and cellular energy reserves, enhancing the activity of silent mating type information regulation 2 homolog 1 (SIRT1) by elevating intracellular nicotinamide adenine dinucleotide (NAD+) levels, consequently leading to sustained activation of PGC1α through this signaling cascade (38). As a central regulator of mitochondrial biogenesis and function, PGC-1α has demonstrated significant therapeutic potential as a target in the management of various metabolic liver diseases (39). Furthermore, PGC1α positively regulates the transcriptional activity of nuclear respiratory factor 1 (NRF1) and mitochondrial transcription factor A (TFAM) (40), with the triad synergistically increasing mitochondrial DNA (mtDNA) copy number and maintaining mitochondrial homeostasis (41). This aligns with our findings that FSP intervention significantly upregulates AdipoR1, AdipoR2, and phosphorylated AMPK (p-AMPK) levels in mouse liver tissues and FSP serum-treated cells, while effectively promoting the expression of the downstream key factor PGC-1α. Accompanying this regulatory process, the transcriptional levels of NRF1 and TFAM were markedly elevated, and mtDNA copy number concurrently increased, indicating effective activation of the PGC1α/NRF1/TFAM signaling axis.

Previous studies have indicated that cytochrome c oxidase subunit IV (COXIV) can serve as a marker for assessing mitochondrial function (42), with its expression level showing a significant positive correlation with PGC1α. However, there is currently a lack of evidence demonstrating a clear and direct regulatory effect of PGC1α on COXIV. Nevertheless, some studies suggest that PGC1α may indirectly modulate COXIV expression through the NRF-1/TFAM signaling axis (43). In this study, we observed a marked upregulation of COXIV protein expression in liver tissues and HepG2 cells, along with increased mRNA levels of NRF1 and TFAM following FSP intervention. This suggests that FSP may promote the synthesis of mitochondrial functional proteins such as COXIV by activating the PGC-1α-dependent NRF1/TFAM signaling pathway.

Additionally, PGC1α plays a pivotal role in the regulation of lactate metabolism (44). Studies have demonstrated that lactate accumulation in skeletal muscle constitutes one of the key factors inducing exercise-induced fatigue (45). Under physiological conditions, lactate produced in muscles is primarily metabolized and cleared in the liver via the Cori cycle (46). Lactate metabolism involves a key enzyme—lactate dehydrogenase (LDH), which forms homologous or heterotetrameric complexes through different combinations of two subunits, LDHA and LDHB. Among them, lactate dehydrogenase subunit B (LDHB) (47) effectively reduces systemic lactate levels by converting lactate into pyruvate. Notably, LDHB is localized in mitochondria, and its expression is directly regulated by PGC1α (48). To investigate whether the lactate-regulating capacity of FSP is associated with LDHB, this study conducted validation at the protein level. The results demonstrated that LDHB expression was significantly upregulated in liver tissues and HepG2 cells treated with FSP, confirming that FSP may promote lactate metabolism and alleviate exercise-induced fatigue through the PGC1α/LDHB pathway.

Current studies indicate that AdipoR1 primarily participates in the activation of the AMPK signaling pathway, whereas AdipoR2 tends to function through the PPARα pathway (49). However, in this study, although no significant changes in PPARα expression were observed, the expression of two key genes involved in fatty acid β-oxidation (50) - carnitine palmitoyltransferase 1α (CPT1α/CPT1a) and carnitine palmitoyltransferase 1β (CPT1β/CPT1b)—showed an upward trend, suggesting that FSP may regulate lipid metabolism through a PPARα-independent pathway. Previous literature has reported that AMPK can promote fatty acid oxidation and reduce lipid accumulation by inhibiting the activity of acetyl-CoA carboxylase (ACC), thereby decreasing the allosteric inhibition of CPT1 by its product, malonyl-CoA (51). This may represent a key molecular mechanism by which AMPK regulates lipid metabolism in FSP, but further exploration is warranted. To clarify the necessity of AMPK in FSP-mediated biological effects, this study employed an AMPK inhibitor (Compound C) to establish a pathway inhibition model at the cellular level. Results showed that upon AMPK inhibition, the expression levels of its downstream key molecules (including SIRT1, PGC1α, NRF1, TFAM, COXIV, and LDHB) were significantly downregulated, accompanied by a trend of decreased ATP production, significantly elevated ROS levels, markedly reduced mtDNA copy number, and a substantial decrease in mitochondrial membrane potential. Notably, in the FSP serum-treated groups, the extent of inhibition in these indicators was alleviated compared to the vehicle group, indicating that FSP exerts a certain protective effect on the AMPK signaling pathway and its mediated mitochondrial function. Additionally, transmission electron microscopy (TEM) analysis confirmed that cells cultured with FSP serum exhibited an increase in mitochondrial number and cross-sectional area, along with enriched cristae structure density. However, these morphological improvements were significantly reversed when combined with the AMPK inhibitor. These findings further validate the central role of AMPK in FSP-mediated regulation of mitochondrial biogenesis and function at the structural level.

FSP also exerts anti-fatigue effects through multiple parallel pathways. Fatigue in the organism is often accompanied by elevated oxidative stress levels and exacerbated inflammatory responses (52). In terms of antioxidation, this study demonstrated that FSP intervention significantly upregulated the expression of nuclear factor E2-related factor 2 (NRF2) and its downstream target heme oxygenase-1 (HO-1) in mouse liver tissues and HepG2 cells. Given that AMPK activation can positively regulate the NRF2/HO-1 pathway (53), it is suggested that FSP may exert antioxidant effects through the AMPK signaling cascade. Regarding anti-inflammatory effects, FSP significantly downregulated the protein expression levels of cyclooxygenase-2 (COX2) and tumor necrosis factor-α (TNFα), preliminarily confirming its anti-inflammatory role. Further differential gene expression analysis revealed that the expression of tumor necrosis factor receptor superfamily member 1B (Tnfrsf1b/TNFR2) and nuclear factor κB inhibitor protein α (Nfkbia/IκBα) was significantly upregulated in liver tissues of the FSP intervention group. As an important receptor for TNF, TNFR2 exerts inhibitory effects on inflammatory progression through its signal transduction (54), while IκBα can prevent nuclear translocation of NF-κB by binding to it, thereby inhibiting the expression of downstream inflammatory factors (55, 56). Although both *Smilax glabra* Roxb. and *Ficus hirta* Vahl. individually have been reported to possess anti-inflammatory and antioxidant activities (7, 8), the synergistic upregulation of TNFR2 and IκBα by FSP suggests that it may enhance anti-inflammatory effects by modulating the TNF/TNFR2 and IκBα/NF-κB signaling axes, providing a new direction for further analysis of the anti-inflammatory mechanism of FSP.

Unlike traditional studies confined to the investigation of single herbal medicines, this research focuses on the pharmacodynamic material basis of folk traditional herbal compound formulas. In the long-term practice and application of traditional Chinese medicine, compound formulas exert holistic effects through the synergistic combination of multiple medicinal herbs (57). After confirming the significant physiological regulatory effects of FSP, we established a multi-parameter evaluation system and ultimately identified taxifolin and apigenin as the potential primary active components responsible for FSP's biological activities. However, it should be noted that FSP has a complex composition, and its overall efficacy is inevitably mediated by multiple active substances. Although some components exhibited favorable binding potential with AdipoRs, they were not ultimately selected due to factors such as low oral bioavailability, limited gastrointestinal absorption efficiency, or low content. Nevertheless, this does not exclude the potential roles of other active components in activating AdipoRs and their downstream pathways. Subsequent studies could further validate these effects through monomer purification combined with *in vivo*/*in vitro* experiments.

Regarding apigenin, its *in vivo* metabolic processes primarily involve hepatic biotransformation and gut microbiota-mediated modifications (58). Studies have demonstrated that apigenin can elevate adiponectin levels and activate AdipoRs (59). Our experimental results further support this conclusion. Molecular docking analysis revealed strong binding capacity between apigenin and AdipoRs, particularly with AdipoR1, exhibiting a binding free energy as low as−10.2 kcal/mol, indicating high affinity. As a quality control marker for *Ficus hirta* Vahl., apigenin is abundant in this herb (26) and is also detectable in *Smilax glabra* Roxb. (7). Research has confirmed that apigenin participates in lipid metabolism regulation by upregulating CPT1 expression (60) and exhibits definite antioxidant and anti-inflammatory activities (61). Notably, this compound significantly increases intracellular NAD+ levels (58), suggesting its potential involvement in energy metabolism regulation via activation of the NAD+-dependent deacetylase SIRT1. This mechanism was validated in our results: apigenin intervention significantly upregulated SIRT1 expression and its downstream key coactivator PGC1α in cells, thereby enhancing mitochondrial biogenesis and energy metabolism efficiency at the functional level. Although oral bioavailability of apigenin remains somewhat limited, its long-standing traditional medicinal use confirms its safety (62). Particularly with the development of novel carbon nanopowder solid dispersion carrier systems, its relative oral bioavailability has been increased by approximately 183% (63). This breakthrough provides a crucial foundation for subsequent research and related formulation development.

Molecular docking analysis of taxifolin demonstrated that it also exhibited superior binding affinity for AdipoR1 compared to AdipoR2, with a binding free energy of−10.2 kcal/mol. As one of the most biologically active representatives in the flavonoid family (64), taxifolin exhibits favorable safety profiles (65). The C2–C3 double bond configuration in its molecular structure critically determines its hepatoprotective activity (66). Studies have shown that taxifolin is an important active component of silymarin, a commonly used clinical hepatoprotective drug (67). It significantly reduces serum levels of ALT and AST (68), demonstrates potential in regulating hepatic lipid metabolism (65), and possesses both antioxidant and anti-inflammatory properties (69). Although direct studies on the association between taxifolin and the adiponectin system remain limited, existing literature reports that this compound can elevate serum adiponectin levels in type 2 diabetic animal models (70). Building on these findings, our study further clarified that taxifolin effectively activates the AdipoRs-AMPK signaling axis, thereby regulating downstream energy metabolic networks. However, taxifolin exhibits relatively low stability and membrane permeability (71), necessitating the development of suitable drug delivery systems for practical applications. Further research is also required to elucidate its metabolic transformation patterns in the human body and the biological effects of its active metabolites.

Drug-target protein interactions represent a critical step in exerting pharmacological effects (72). To further validate the regulatory effects of apigenin and taxifolin on AdipoRs and their downstream pathways, this study employed the Cellular Thermal Shift Assay (CETSA) to assess the binding characteristics of these two active components to AdipoRs. Experiments confirmed that both taxifolin and apigenin effectively bind to AdipoRs, with higher affinity for AdipoR1 than AdipoR2. Moreover, they replicated the biological activities of FSP *in vitro* models: both compounds enhanced mitochondrial function and promoted fatty acid β-oxidation by modulating the AdipoRs-AMPK axis. Furthermore, based on the multi-component synergy theory (73) and inspired by studies demonstrating that the combination of apigenin and quercetin significantly improves mitochondrial function and promotes adiponectin secretion (74), this study investigated the combined intervention of taxifolin (also known as dihydroquercetin) and apigenin. Experimental results indicated that the co-intervention of these two components significantly activated the AdipoRs-AMPK signaling pathway and exhibited superior synergistic effects across multiple parameters compared to individual treatments, suggesting complementary or synergistic interactions in regulating energy metabolism pathways.

This experiment also has certain limitations. First, the primary objective of the experiment is to evaluate the physiological effects of FSP while excluding confounding factors such as the gavage procedure. However, to further enhance the reliability and rigor of the research findings, future studies should consider incorporating an untreated sedentary control group, as well as a positive control group treated with a known anti-fatigue agent. Moreover, the combination of *Smilax glabra* Roxb. and *Ficus hirta* Vahl. originates from traditional folk experience, and there is currently no definitive literature documenting its standard ratio. The ratio selected for this experiment was based on a commonly used folk proportion identified through preliminary literature screening, and subsequent experiments confirmed that this ratio produces favorable effects. However, whether this ratio represents the optimal compatibility remains unclear, and further systematic research is needed to determine the most effective proportion. Secondly, it should be noted that drug dosage is significantly influenced by factors such as body weight. Although the dosage administered to mice in this study was converted based on a human body weight of 50 kg, due to substantial differences in body weight distribution between Eastern and Western populations, whether this dosage is applicable to other populations requires further in-depth investigation. Furthermore, although it reveals the significant role of FSP in anti-fatigue and improving exercise performance, and preliminarily identifies key active components involved in these biological effects, there is room for improvement in the research methodology. The current work has not yet employed quantitative analytical techniques such as high-performance liquid chromatography (HPLC) or liquid chromatography-mass spectrometry (LC-MS) to precisely determine the specific content of each compound in FSP. Instead, it relied solely on literature and database screening to identify important components from the extracts of *Smilax glabra* Roxb. and *Ficus hirta* Vahl., which limits the in-depth exploration of the correspondence between monomer dosages and the effective dose of the compound formula. It is important to note that reported component concentrations in herbs vary significantly across different studies. Apart from astilbin as the most abundant component in *Smilax glabra* Roxb. and the primary quality control markers in *Ficus hirta* Vahl. (psoralen, apigenin, bergapten) (26) being relatively consistent, the selection of other components was based on compounds commonly reported at relatively high levels across various literature sources (listed without ranking priority). Notably, since this study used a formulated preparation rather than individual herbs, and some active components overlap between the two herbs, the precise ratio of each component in FSP and their synergistic mechanisms require further clarification through more systematic quantitative analysis in future research.

## 5 Conclusion

In summary, this study is the first to reveal and elucidate the molecular mechanism by which the *Smilax glabra* Roxb. and *Ficus hirta* Vahl. aqueous extract (FSP) enhances exercise performance through the core AdipoRs-AMPK signaling pathway: FSP activates AdipoR1 and AdipoR2 receptors, initiating the AMPK signaling cascade, which synergistically regulates multiple key networks - enhancing mitochondrial biogenesis and function via the PGC1α/NRF-1/TFAM axis, promoting lactate metabolism and clearance through the PGC1α/LDHB pathway, facilitating fatty acid β-oxidation by upregulating CPT1a/b expression, and activating the NRF2/HO-1 antioxidant pathway while suppressing NF-κB-mediated inflammatory responses. Furthermore, by establishing a multi-parameter evaluation system, we preliminarily identified taxifolin and apigenin as potential core active components of FSP. Both compounds exhibit high affinity for AdipoRs, and CETSA experiments confirmed their direct binding capacity. Notably, their combination demonstrated synergistic activation effects. These findings not only provide new molecular evidence for the multi-component synergistic actions of natural products but also establish a solid theoretical foundation for developing standardized exercise nutritional supplements targeting the AdipoRs-AMPK pathway. This work holds significant application value for advancing the precise utilization of medicinal and edible plants and promoting the clinical translation of multi-target anti-fatigue agents.

## Data Availability

The raw data generated in this study can be found in the NCBI BioProject repository under accession PRJNA1471468: http://www.ncbi.nlm.nih.gov/bioproject/1471468.
