# Supplementary material for: The herbal pair of Smilax glabra Roxb. and Ficus hirta Vahl. improves exercise performance by regulating mitochondrial function via the adiponection receptors-mediated AMPK signaling pathway
Source: Front Nutr. 2026 Jun 15;13:1782001. doi: 10.3389/fnut.2026.1782001 (PMC13312815; doi:10.3389/fnut.2026.1782001)
Supplement: Supplementary file 1 [file Supplementary_file_1.docx]

Supplementary Material

# Supplementary Data

Figure S1


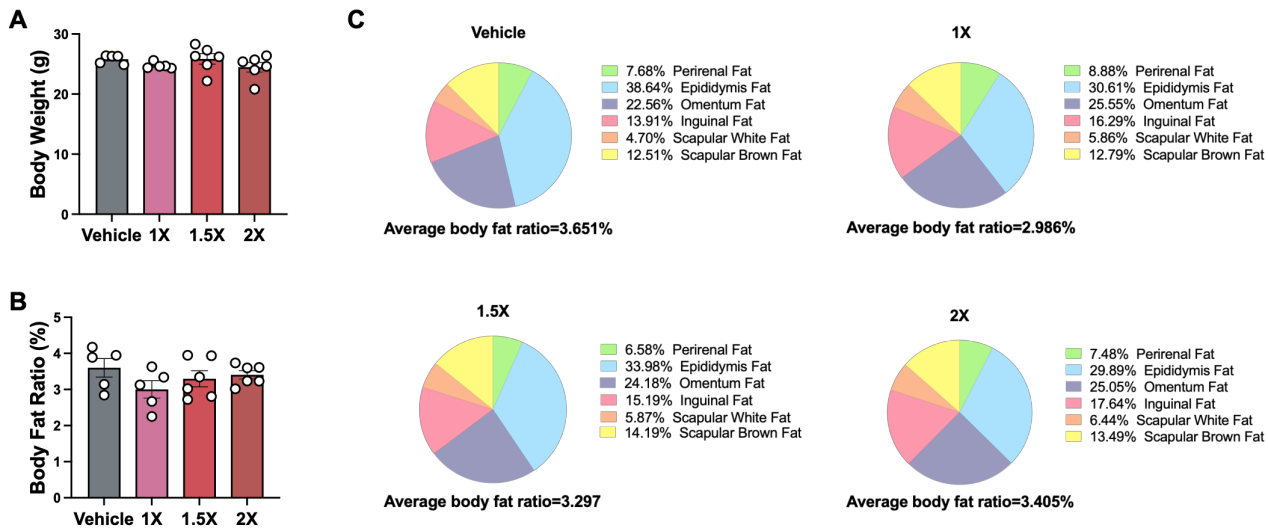


Fig S1. Baseline data collection in FSP group mice before the experiment

1. B) Body weight and body fat rate statistics of mice before the experiment in the FSP group (n=5); (C) Average body fat rate of each fat in experimental mice in the FSP group. Results are expressed as mean ± SEM.

Figure S2


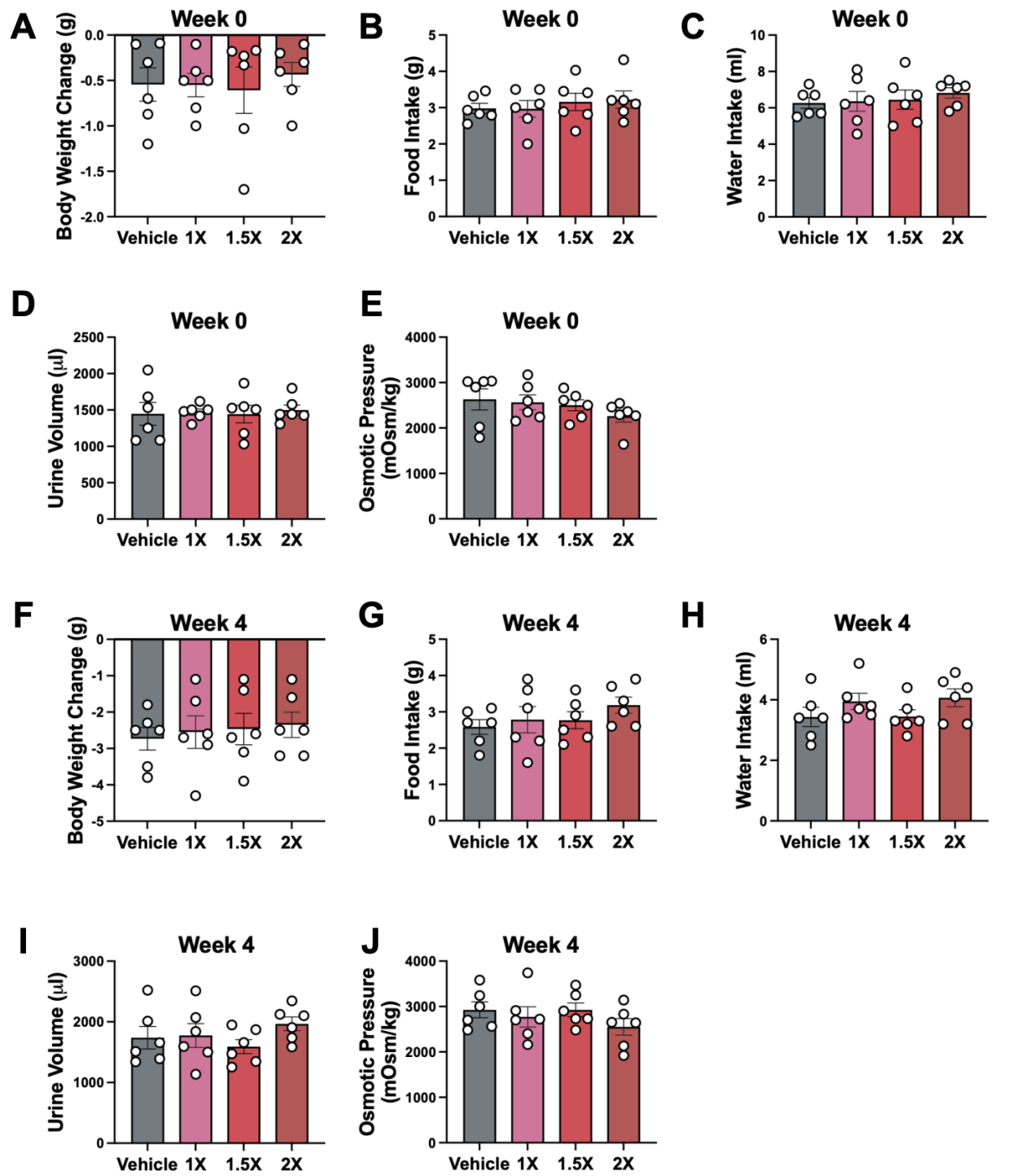


Fig S2. Collection of 24-hour metabolic cage data from FSP group mice before the experiment and after gavage

(A-E) Collection of basal metabolic data from mice in the FSP group before the experiment (24-hour metabolic cage, n=6): A-Body weight changes in mice; B-Food intake in mice; C-Water intake in mice; D- Urine osmotic pressure statistics; E-Urine osmolality in mice; (F-J) Collection of basal metabolic data from mice in the FSP group after gavage (24-hour metabolic cage, n=6): F-Body weight changes in mice; G-Food intake in mice; H-Water intake in mice; I-Urine osmotic pressure statistics; J-Urine osmolality in mice. Results are expressed as mean ± SEM.

Figure S3


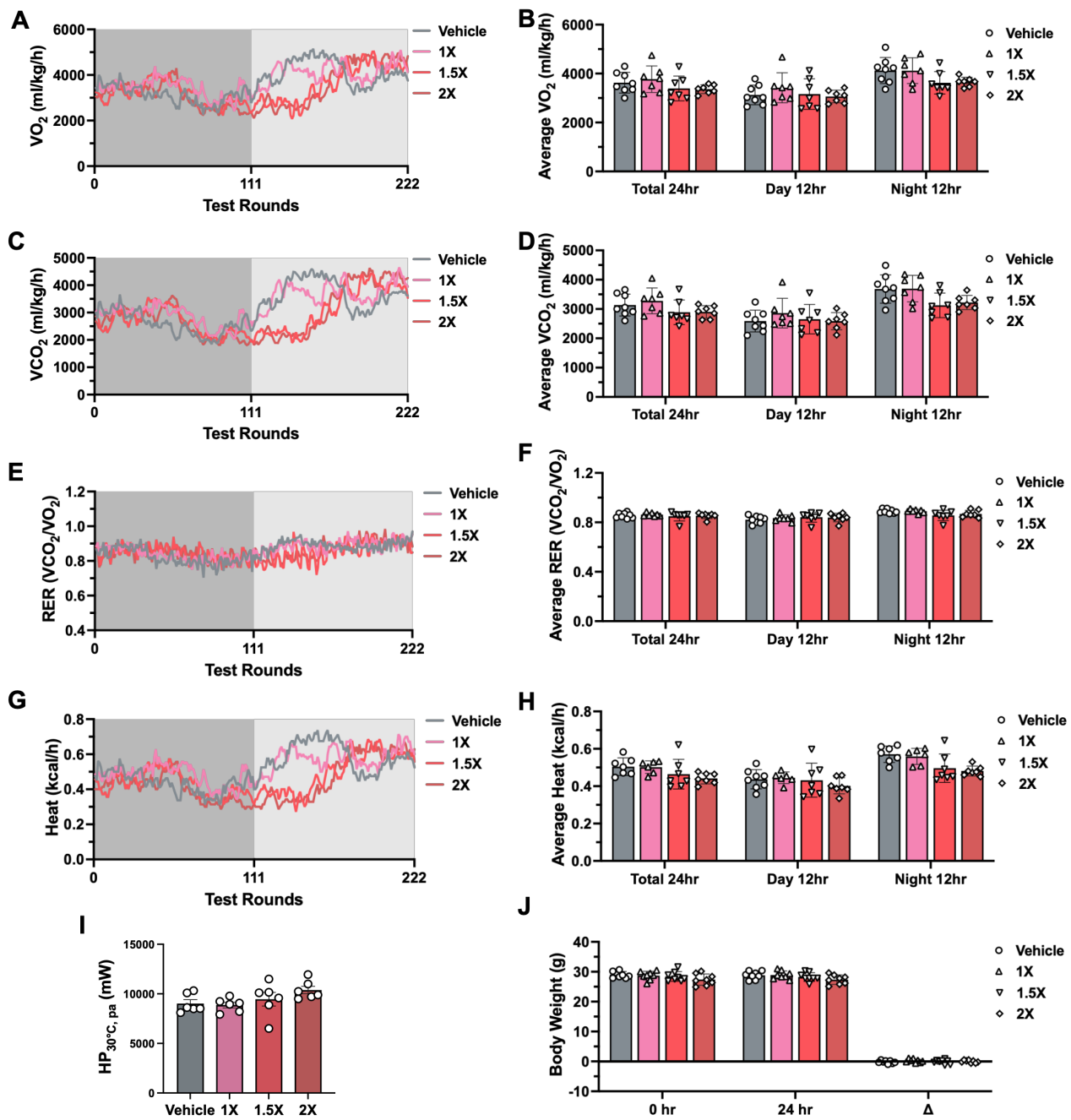


Fig S3. Metabolic monitoring data of FSP group mice before the experiment

(A-F) Oxygen consumption (VO2), carbon dioxide production (VCO2), and respiratory exchange ratio (RER) in FSP group mice before the experiment (n=8); (G-H) Heat production in mice before the experiment (n=7); (I) Basal metabolic rate (BMR) in mice before the experiment under 30℃ environmental conditions (n=6); (J) Body weight records of mice during metabolic monitoring before the experiment (n=8). Results are expressed as mean ± SEM.

Figure S4


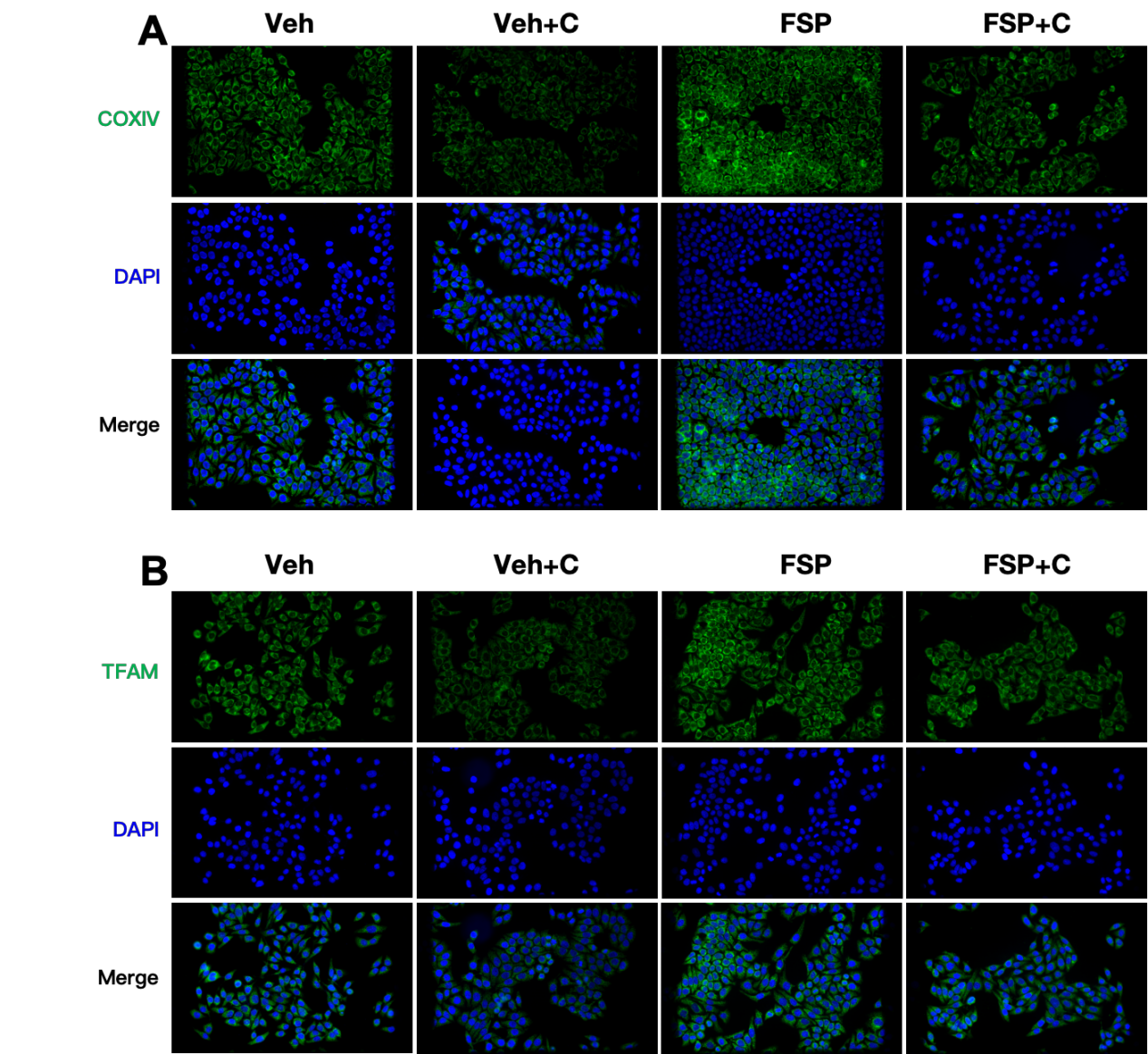


Fig S4. Effect of AMPK inhibitor on the expression of mitochondrial-related genes

(A-B) Immunofluorescence of COXIV and TFAM in HepG2 cells treated with AMPK inhibitor (Compound C) in Vehicle serum group and FSP serum group (Scale bar: 20μm).

Figure S5


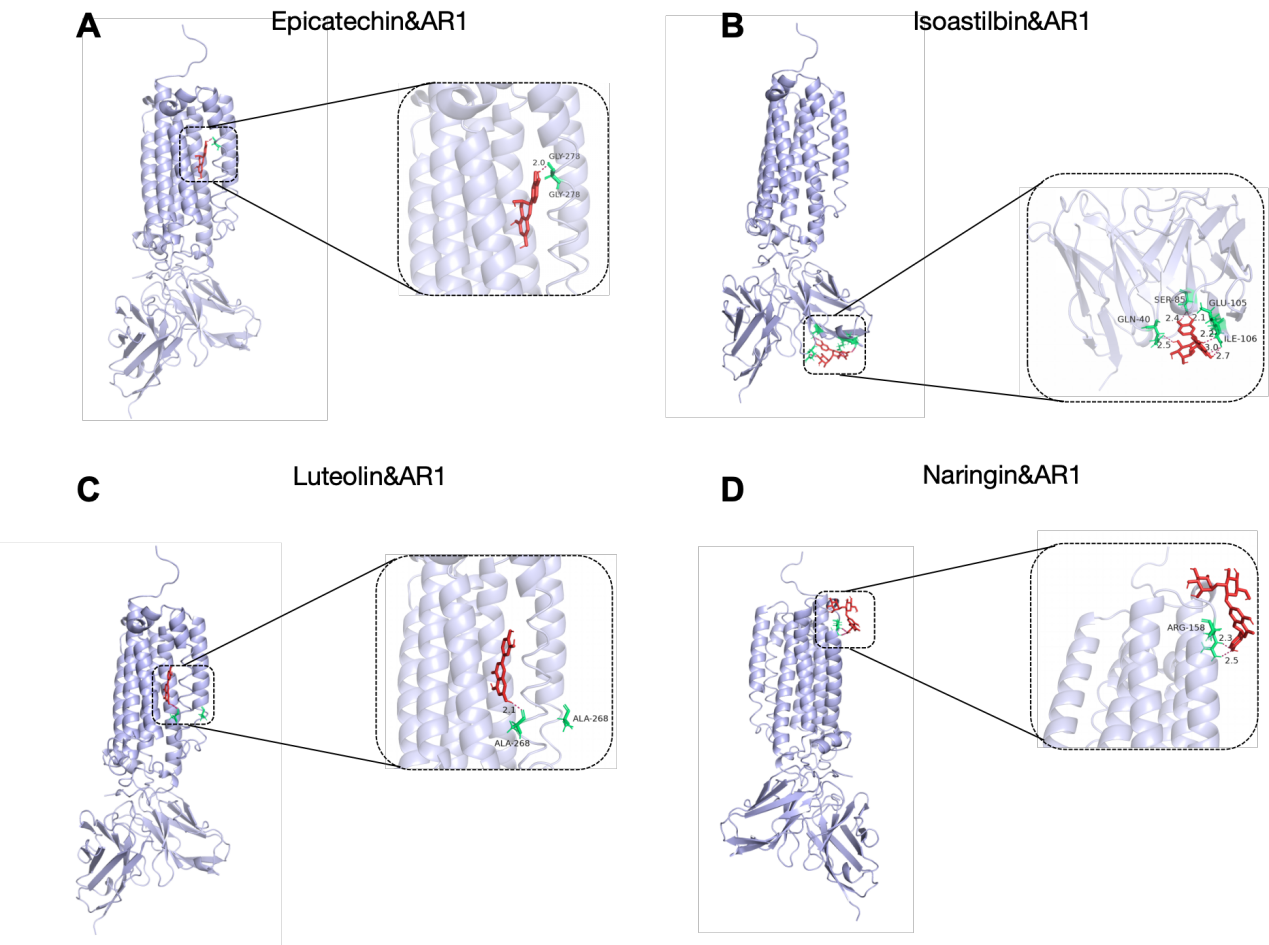


Fig S5. Prediction of targets between potential active ingredients in FSP and AdipoR1

(A)Molecular docking analysis of Epicatechin with AdipoR1 using AutoDock; (B) Molecular docking analysis of Isoastilbin with AdipoR1 using AutoDock; (C) Molecular docking analysis of Luteolin with AdipoR1 using AutoDock; (D) Molecular docking analysis of Naringin with AdipoR1 using AutoDock.

Figure S6


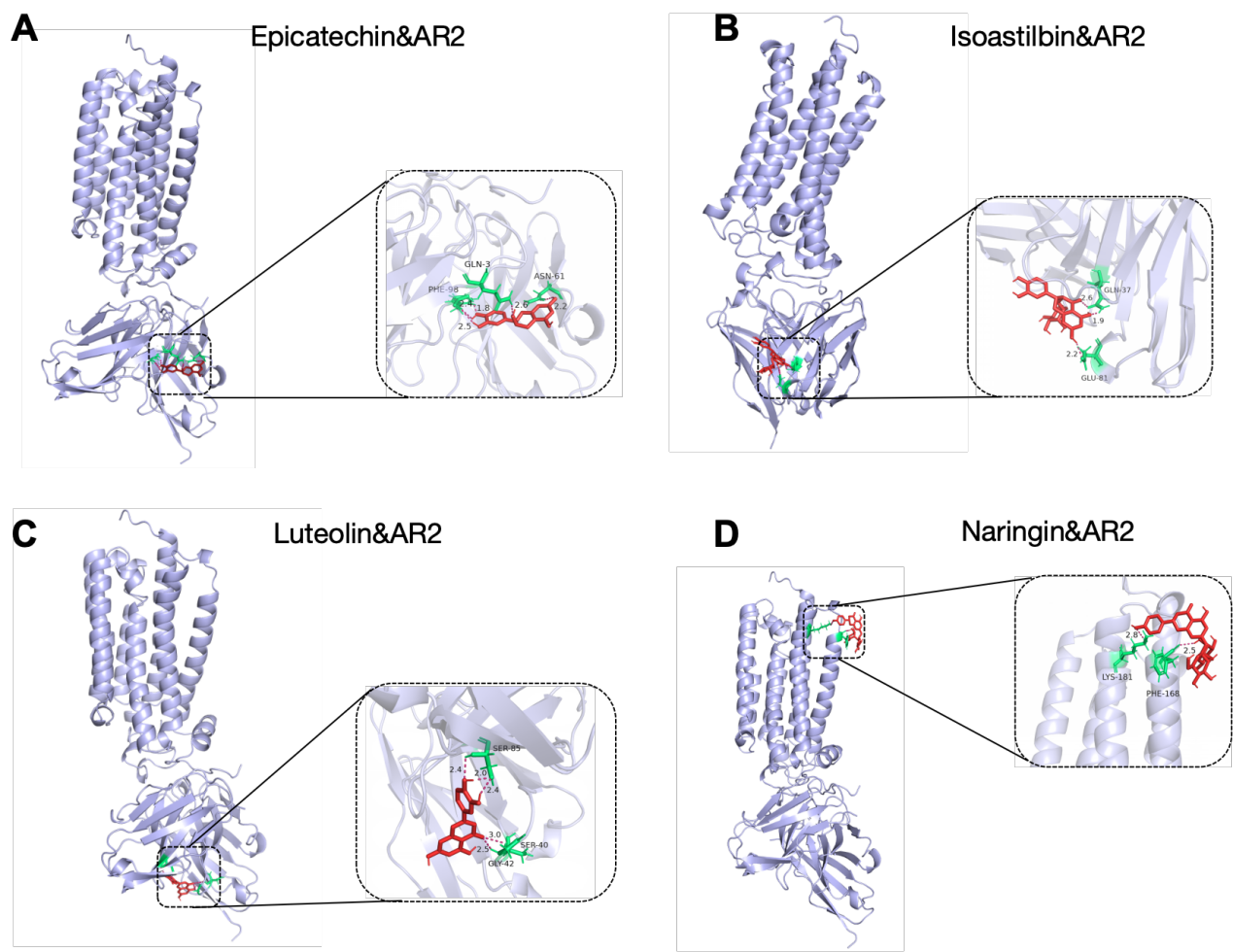


Fig S6. Prediction of targets between potential active ingredients in FSP and AdipoR2

(A)Molecular docking analysis of Epicatechin with AdipoR2 using AutoDock; (B) Molecular docking analysis of Isoastilbin with AdipoR2 using AutoDock; (C) Molecular docking analysis of Luteolin with AdipoR2 using AutoDock; (D) Molecular docking analysis of Naringin with AdipoR2 using AutoDock.

Figure S7


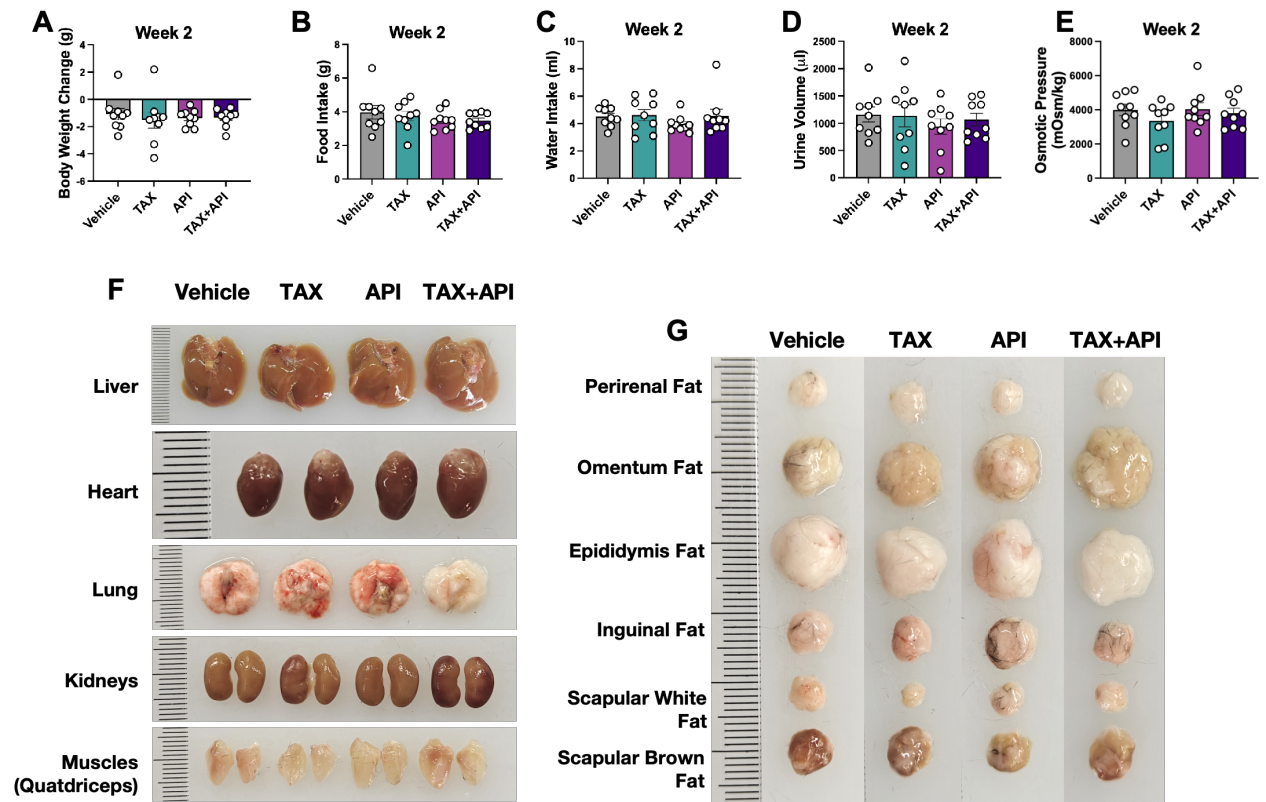


Fig S7. Collection of Basic parameters in the monomer group mice before the experiment

(A-E) Collection of basal metabolic data from mice in the monomer group before the experiment (24-hour metabolic cage, n=9): A-Body weight changes in mice; B-Food intake in mice; C-Water intake in mice; D-Urine osmotic pressure statistics; E-Urine osmolality in mice; (F-G) Organ and adipose tissue observations in monomer group. Results are expressed as mean ± SEM.

Figure S8


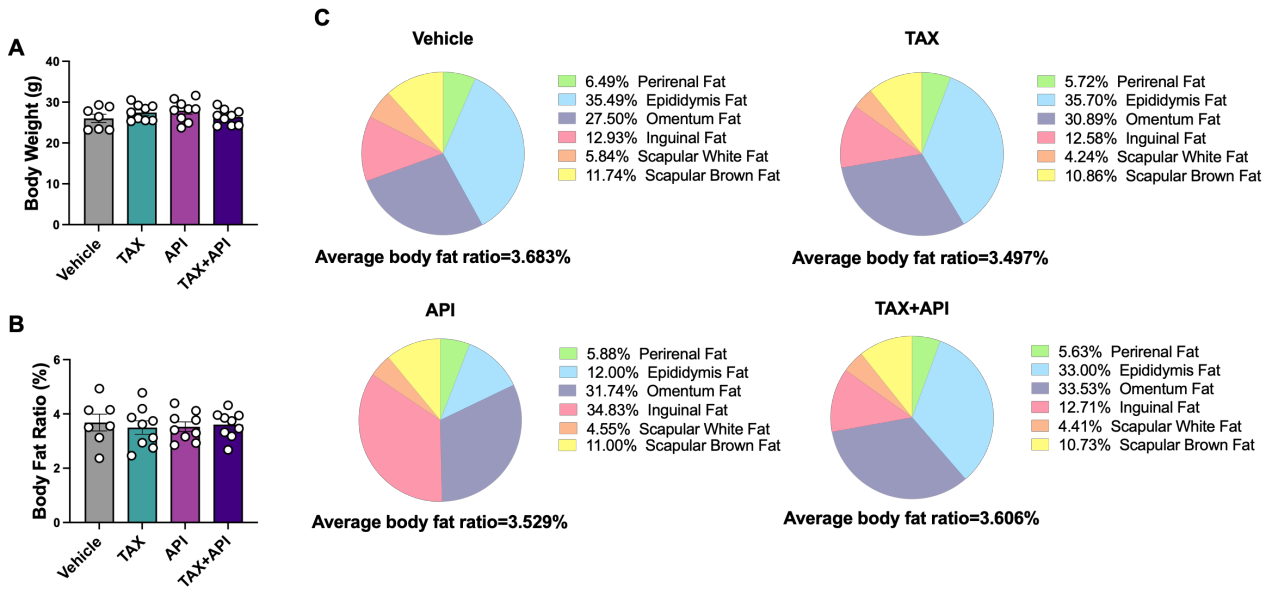


Fig S8. Statistical analysis of body weight and body fat rate in the monomer group mice before the experiment

(A-B) Body weight and body fat rate statistics of mice before the experiment in the monomer group (n=5); (C) Average body fat rate of each fat in experimental mice in the monomer group. Results are expressed as mean ± SEM.

Figure S9


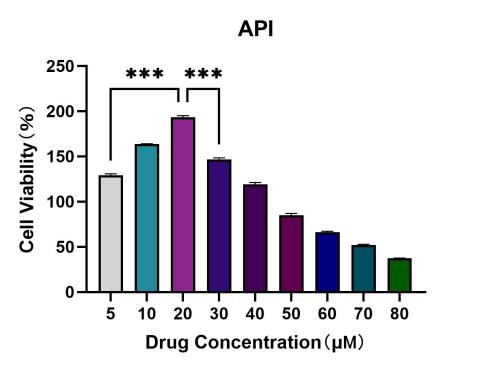

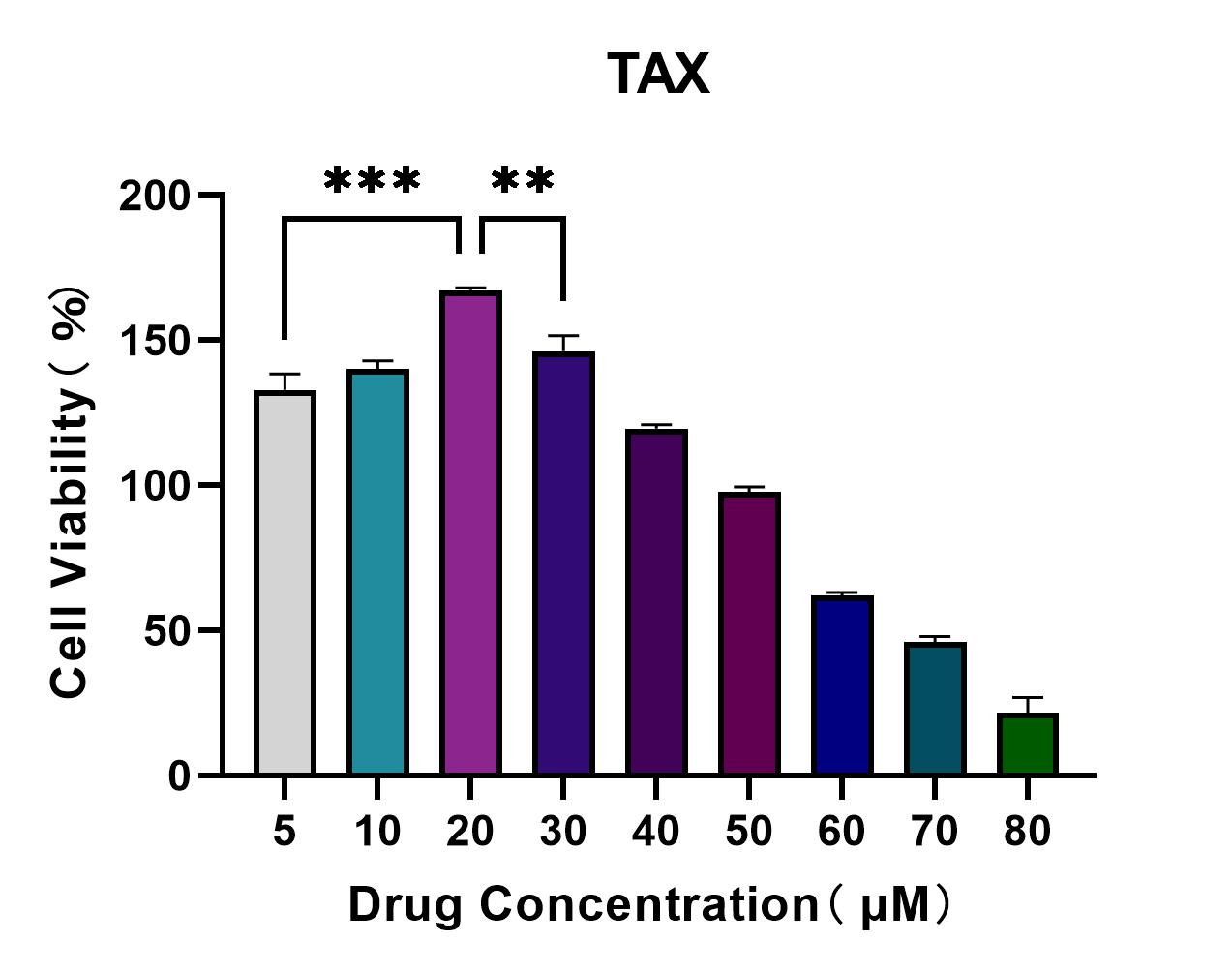

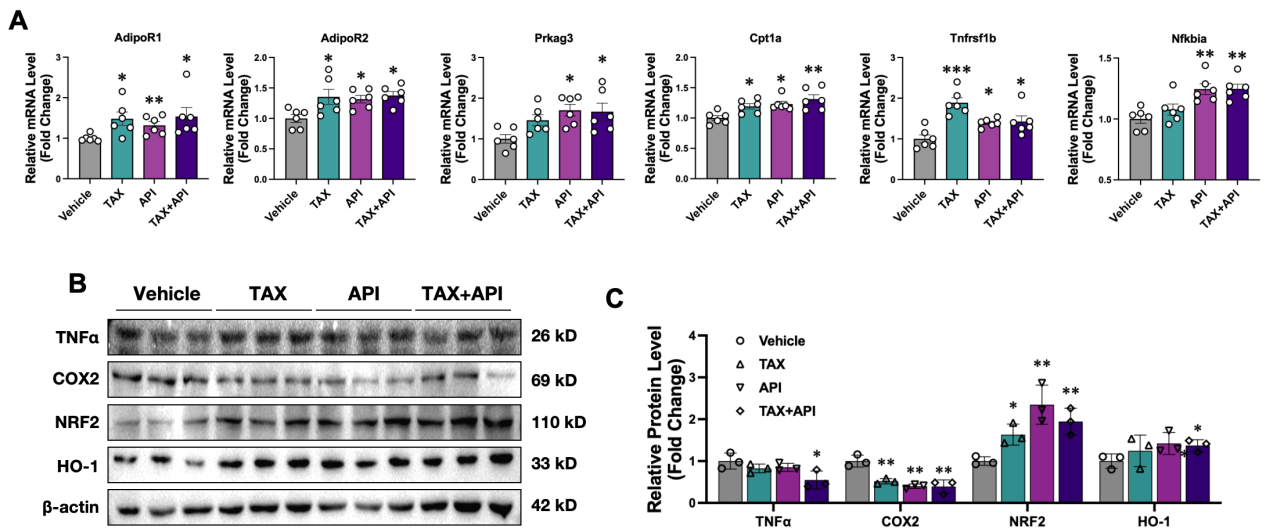


**E**

**D**

Fig S9. Taxifolin and apigenin activate the AdipoRs, exerting anti-inflammatory and antioxidant effects.

(A)Differential gene mRNA expression in the liver of the monomer group mice (n=6); (B-C) Expression and semi-quantitative analysis of pro-inflammatory and antioxidant proteins in the liver tissue of the monomer group mice(n=3). (D)Taxifolin-CCK8; (E) Apigenin-CCK8.Results are expressed as mean ± SEM. **p<0.*05, ***p*<0.01, ****p*<0.001 compared to the Vehicle group.

# Supplementary Tables

Table S1. The information of primers used in the study

| Gene | Sequence (5’ to 3’) | |
| --- | --- | --- |
|  | Forward | Reverse |
| mAdipoR1 | AATGGGGCTCCTTCTGGTAAC | GGATGACTCTCCAACGTCCCT |
| hAdipoR1 | CCTGGAAAATTTGACATATGGTTC | AGGCTCAGAGAAGGGTGTCA |
| mAdipoR2 | GCCAAACACCGATTGGGGT | GGCTCCAAATCTCCTTGGTAGTT |
| hAdipoR2 | CGGGGAGTAAGAGCAGGAG | GGGCAGCTCCTGTGATGTAG |
| mPrkag3 | CTGAGTTGGGATGACGAACTTC | GATGACCAATTTGGAGCTGGTA |
| hPrkag3 | CCAGAGGCACTAGGATTACC | TCTCTTGCCACAAGTTCAGG |
| mSirt1 | TGATTGGCACCGATCCTCG | CCACAGCGTCATATCATCCAG |
| hSirt1 | AGTGGCAAAGGAGCAGATTAG | CTGCCACAAGAACTAGAGGATAAG |
| mPpargc1a | CCCTGCCATTGTTAAGACC | TGCTGCTGTTCCTGTTTTC |
| hPpargc1a | AGACTATTGAACGCACCTTA | CCTTTCTTGGTGGAGTTATT |
| mCpt1a | TGGCATCATCACTGGTGTGTT | GTCTAGGGTCCGATTGATCTTTG |
| hCpt1a | AGGACGCTACTTCAAGGTCTGG | CGTATCCGGGTCTTCACTTCTGT |
| mCpt1b | GACTTCCGGCTTAGTCGGG | GAATAAGGCGTTTCTTCCAGGA |
| hCpt1b | CTCCTTTCCTTGCTGAGGTG | GCGTTCGTCTCTGAGCTTG |
| mTnfrsf1b | ACACCCTACAAACCGGAACC | AGCCTTCCTGTCATAGTATTCCT |
| hTnfrsf1b | AATGCCGTCCAGCATACAGT | CAGTCCTGGGAGAACCTCAG |
| mNfkbia | TGAAGGACGAGGAGTACGAGC | TGCAGGAACGAGTCTCCGT |
| hNfkbia | TCAATGCTCAGGAGCCCTGTAAT | AGCC-CTGGTAGGTAACTCTGT |
| mNRF1 | AGCACGGAGTGACCCAAAC | AGGATGTCCGAGTCATCATAAGA |
| hNRF1 | AATTATTCTGCCGTGGCTGATGG | GATGCTTGCGTCGTCTGGATG |
| mTFAM | AACACCCAGATGCAAAACTTTCA | GACTTGGAGTTAGCTGCTCTTT |
| hTFAM | TCCAAGAAGCTAAGGGTGATTC | TGGTTTCCTGTGCCTATCCATT |
| mβ-actin | AGCCATGTACGTAGCCATCC | GCTGTGGTGGTGAAGCTGTA |
| hβ-actin | CGACAGGATGCAGAAGGAGAT | CAAGAAAGGGTGTAACGCAACTA |

Table S3. General characteristics of mice with 2-week Taxifolin (TAX) and/or Apigenin (API) administration

|  | Vehicle | FSP-1X | FSP-1.5X | FSP-2X | Trend Analysis |
| --- | --- | --- | --- | --- | --- |
| Body Weight (g) | 25.82±0.7120 | 24.72±0.5167 | 25.87±2.129 | 24.48±1.990 | *p*=0.3520 |
| Body Length (cm) | 17.56±0.2191 | 17.06±0.6804 | 17.47±0.2338 | 17.20±0.4940 | *p*=0.2677 |
| Weight (g) | | | | | |
| Liver | 1.212±0.1820 | 1.397±0.08723 | 1.442±0.1419 | 1.319±0.1388 | *p*=0.0762 |
| Left Kidney | 0.1714±0.01401 | 0.1524±0.01558 | 0.1782±0.02037 | 0.1577±0.02173 | *p*=0.1132 |
| Right Kidney | 0.1682±0.01047 | 0.1544±0.01750 | 0.1750±0.02027 | 0.1563±0.02089 | *p*=0.2126 |
| Heart | 0.1294±0.01919 | 0.1232±0.01737 | 0.1457±0.02370 | 0.1322±0.01901 | *p*=0.3235 |
| Lung | 0.1670±0.05948 | 0.1474±0.02942 | 0.1755±0.02892 | 0.1857±0.07672 | *p*=0.6882 |
| Muscles (Quadriceps) | 0.3260±0.02986 | 0.2872±0.02341 | 0.2980±0.03989 | 0.2858±0.02469 | *p*=0.1614 |
| Relative Weight (%) |  |  |  |  |  |
| Liver | 4.704±0.7639 | 5.650±0.3306 | 5.586±0.4541 | 5.389±0.3449 | *p*=0.0255* |
| Left Kidney | 0.6636±0.04700 | 0.6170±0.06719 | 0.6928±0.1020 | 0.6438±0.07177 | *p*=0.4284 |
| Right Kidney | 0.6518±0.04033 | 0.6246±0.06988 | 0.6807±0.09607 | 0.6393±0.07345 | *p*=0.6363 |
| Heart | 0.5010±0.06884 | 0.4986±0.07279 | 0.5708±0.1423 | 0.5400±0.06087 | *p*=0.5417 |
| Lung | 0.6496±0.2411 | 0.5962±0.1216 | 0.6827±0.1382 | 0.7623±0.3244 | *p*=0.6690 |
| Muscles (Quadriceps) | 1.280±0.1147 | 1.223±0.1030 | 1.240±0.1249 | 1.258±0.06350 | *p*=0.8363 |

**p<0.*05 compared to the Vehicle group.

Table S3. General characteristics of mice with 2-week Taxifolin (TAX) and/or Apigenin (API) administration

| Characteristic | Vehicle | TAX | API | TAX+API | Trend Analysis |
| --- | --- | --- | --- | --- | --- |
| Body Weight (g) | 25.73±2.680 | 27.43±1.936 | 27.91±2.619 | 26.34±1.892 | *p*=0.2062 |
| Body Length (cm) | 16.83±0.6777 | 17.28±0.3768 | 17.27±0.5196 | 17.12±0.3153 | *p*=0.2167 |
| Organ Weight (g) |  |  |  |  |  |
| Liver | 1.174±0.3655 | 1.325±0.2221 | 1.317±0.2574 | 1.322±0.2215 | *p*=0.6096 |
| Left Kidney | 0.1683±0.04179 | 0.1884±0.02106 | 0.2006±0.03060 | 0.1985±0.02379 | *p*=0.1304 |
| Right Kidney | 0.1676±0.03421 | 0.1991±0.03791 | 0.2018±0.03465 | 0.1881±0.02514 | *p*=0.1634 |
| Heart | 0.1298±0.02925 | 0.1411±0.01387 | 0.02221 | 0.1389±0.01805 | *p*=0.6289 |
| Lung | 0.2972±0.08490 | 0.3042±0.08641 | 0.3258±0.1067 | 0.3151±0.1116 | *p*=0.6882 |
| Muscles (Quadriceps) | 0.2845±0.1014 | 0.3245±0.07387 | 0.3421±0.06097 | 0.3268±0.08877 | *p*=0.9354 |
| Relative Weight (%) |  |  |  |  |  |
| Liver | 4.484±0.9744 | 4.811±0.5755 | 4.694±0.6118 | 4.992±0.5411 | *p*=0.4960 |
| Left Kidney | 0.6520±0.1338 | 0.6864±0.05790 | 0.7186±0.08594 | 0.7521±0.05347 | *p*=0.1224 |
| Right Kidney | 0.6506±0.1169 | 0.7263±0.1343 | 0.7215±0.09345 | 0.7132±0.06898 | *p*=0.4454 |
| Heart | 0.5008±0.07992 | 0.5143±0.03695 | 0.5085±0.04890 | 0.5263±0.04604 | *p*=0.7998 |
| Lung | 1.158±0.3278 | 1.115±0.3244 | 1.186±0.4211 | 1.201±0.4280 | *p*=0.9662 |
| Muscles (Quadriceps) | 1.088±0.3133 | 1.180±0.2416 | 1.227±0.1986 | 1.231±0.2814 | *p*=0.6556 |

**p<0.*05 compared to the Vehicle group.

Table S4. Effect of Taxifolin (TAX) and/or Apigenin (API) on organ, serum and urine biochemical indices

| Parameter | Vehicle | TAX | API | TAX+API |
| --- | --- | --- | --- | --- |
| Organ | | | | |
| Hepatic Glycogen (mg/g) | 38.85±4.591 | 41.28±3.121 | 40.29±8.198 | 48.53±8.317* |
| Hepatic XOD (U/gprot) | 20.00±1.284 | 22.65±2.430 | 22.38±1.897 | 23.15±3.645 |
| Muscle Glycogen  (mg/g) | 4.890±0.2007 | 7.077±2.076 | 6.631±1.636 | 7.132±0.2396*** |
| Serum | | | | |
| AST (U/L) | 83.35±7.585 | 79.82±4.406 | 82.41±4.978 | 78.43±5.559 |
| ALT (U/L) | 43.47±2.264 | 42.43±2.288 | 40.14±4.102 | 40.72±3.898 |
| ALP (U/L) | 4.853±1.674 | 3.769±1.160 | 3.153±1.116 | 4.878±2.604 |
| LDH (U/L) | 385.1±17.19 | 283.7±43.99** | 279.7±43.04** | 272.8±69.35** |
| CK (U/L) | 343.8±54.76 | 200.0±102.5* | 266.6±143.0 | 218.8±50.03** |
| TP (μg/ml) | 1074±29.43 | 1114±74.34 | 1136±81.32 | 1155±97.01 |
| TC (mmol/L) | 3.539±0.3616 | 3.729±0.3011 | 3.436±0.4422 | 3.413±0.3509 |
| TG (mmol/L) | 1.124±0.3678 | 1.148±0.3388 | 0.8996±0.1234 | 1.047±0.2012 |
| Glucose (mmol/L) | 11.58±1.162 | 9.694±1.460* | 9.474±2.881 | 8.817±1.861* |
| Creatinine (μmol/L) | 14.06±6.610 | 18.88±6.685 | 13.23±5.580 | 15.89±6.041 |
| BUN (mmol/L) | 7.186±2.213 | 7.378±1.151 | 7.048±1.700 | 6.781±0.8481 |
| UA (μmol/L) | 39.08±8.120 | 34.67±11.96 | 40.23±12.52 | 38.05±2.646 |
| Ammonia (mmol/L) | 6.266±1.206 | 5.999±0.8221 | 6.143±0.7223 | 7.126±1.604 |
| Urine | | | | |
| Creatinine (μmol/L) | 238.9±112.1 | 332.8±62.08 | 384.2±137.0 | 346.8±64.22 |
| UA(μmol/L) | 9.820±3.701 | 9.307±5.329 | 7.510±6.150 | 10.08±5.409 |
| Na+ (mmol/L) | 126.1±35.67 | 128.0±24.01 | 148.1±19.54 | 149.2±35.30 |
| K+ (mmol/L) | 4.323±0.9538 | 4.427±1.215 | 4.066±0.8140 | 3.995±0.3981 |

**p<0.*05, ***p*<0.01, ****p*<0.001 compared to the Vehicle group.
